# The mitochondrial type IB topoisomerase drives mitochondrial translation and carcinogenesis

**DOI:** 10.1038/s41467-018-07922-3

**Published:** 2019-01-08

**Authors:** S. A. Baechler, V. M. Factor, I. Dalla Rosa, A. Ravji, D. Becker, S. Khiati, L. M. Miller Jenkins, M. Lang, C. Sourbier, S. A. Michaels, L. M. Neckers, H. L. Zhang, A. Spinazzola, S. N. Huang, J. U. Marquardt, Y. Pommier

**Affiliations:** 1 Laboratory of Molecular Pharmacology, Developmental Therapeutics Branch, Center for Cancer Research, NIH, National Cancer Institute, Bethesda, Maryland 20892 USA; 2 Department of Clinical and Movement Neurosciences, Institute of Neurology, Royal Free Campus, University College London, London, NW3 2PF UK; 3 Department of Medicine I, Johannes Gutenberg University, Langenbeckstrasse 1, 55131 Mainz, Germany; 4 Equipe MitoLab, Institut MitoVasc, UMR CNRS 6015, INSERM U1083, Universite d’Angers, 49933 Angers, France; 5 Laboratory of Cell Biology, Center for Cancer Research, National Cancer Institute, National Institutes of Health, Bethesda, MD 20892 USA; 6 Urologic Oncology Branch, Center for Cancer Research, National Cancer Institute, National Institutes of Health, Bethesda, MA 20892 USA; 7 Laboratory of Molecular Oncology, Division of Biotechnology Review and Research I, Office of Biotechnology Products, Center for Drug Evaluation and Research, U.S. Food and Drug Administration, Silver Spring, MD 20993 USA

## Abstract

Mitochondrial topoisomerase IB (TOP1MT) is a nuclear-encoded topoisomerase, exclusively localized to mitochondria, which resolves topological stress generated during mtDNA replication and transcription. Here, we report that *TOP1MT* is overexpressed in cancer tissues and demonstrate that TOP1MT deficiency attenuates tumor growth in human and mouse models of colon and liver cancer. Due to their mitochondrial dysfunction, TOP1MT-KO cells become addicted to glycolysis, which limits synthetic building blocks and energy supply required for the proliferation of cancer cells in a nutrient-deprived tumor microenvironment. Mechanistically, we show that TOP1MT associates with mitoribosomal subunits, ensuring optimal mitochondrial translation and assembly of oxidative phosphorylation complexes that are critical for sustaining tumor growth. The TOP1MT genomic signature profile, based on *Top1mt*-KO liver cancers, is correlated with enhanced survival of hepatocellular carcinoma patients. Our results highlight the importance of TOP1MT for tumor development, providing a potential rationale to develop TOP1MT-targeted drugs as anticancer therapies.

## Abstract (web summary)

TOP1MT is a topoisomerase that is localised to mitochondria. Here, the authors show that TOP1MT has a tumor promoting role in hepatocellular carcinoma by supporting mitochondrial translation and that its deficiency limits tumorigenicity.

## Introduction

Cancer metabolism has been equated with Warburg’s observation that cancer cells undergo aerobic glycolysis converting glucose to lactate in the presence of oxygen; therefore, suggesting the plausibility of defective mitochondria in cancer cells^1,2^. However, ongoing research has revisited Warburg’s initial conclusion and highlighted the central role of mitochondria in cancer^3–5^. Numerous studies demonstrate that mitochondria are not only functional in cancer cells but also that tumors depend on mitochondrial respiration^6^. Furthermore, the cellular functions of mitochondria expand beyond energy production, encompassing redox homeostasis, promotion of cell death, and supply of biosynthetic metabolism^4,7,8^. Based on their implication in multiple cellular functions, mitochondria empower cancer cell growth and survival in the pathological tumor microenvironment by ensuring biosynthetic and bioenergetics supply^6^. Hence, developing therapeutic interventions specifically targeting mitochondria and mitochondria-associated signaling pathways has emerged as a potential approach to suppress tumor growth^3,9^.

Topoisomerases are ubiquitous enzymes required to relax DNA supercoiling and remove the intertwining of DNA molecules such as knots and catenanes^10^. During replication, transcription, chromatin remodeling, or chromosomal segregation, torsional stress occurs as the DNA is constrained due to the highly organized and packed state of chromatin^11,12^. Topoisomerases release topological stress whilst introducing transiently enzyme-linked DNA strand breaks^10,13^. Although the controlled cleavage is required to ensure topological homeostasis, aberrant topoisomerase activity represents a constant threat to the genome^10,13^. Antibacterial and anticancer drugs stabilizing the covalent enzyme-DNA-complex are referred to as topoisomerase poisons, converting these essential enzymes into DNA damaging proteins^13–15^. The accumulation of DNA breaks and protein crosslinks ultimately leads to cell death, which is the mode of action of clinically used topoisomerase inhibitors^13–16^.

Among the six vertebrate topoisomerases, TOP1MT is the only topoisomerase exclusively devoted to mitochondria^10,17^. Mitochondria contain their own circular DNA (mtDNA) encoding 13 proteins of the oxidative phosphorylation complexes. TOP1MT has been shown to be critical for the maintenance of mtDNA integrity and for limiting mtDNA negative supercoiling^18^. In contrast to other topoisomerases, TOP1MT is dispensable in mice with no obvious phenotype of TOP1MT knockout mice under basal conditions^18,19^. However, in a liver regeneration model, TOP1MT deficiency attenuates hepatocyte proliferation by limiting mtDNA expansion. This indicates that TOP1MT may become a limiting factor for highly proliferating cells^20^. Accordingly, depletion of mtDNA impacts cellular proliferation resulting in delayed tumorigenesis^21^. As *TOP1MT* is strongly upregulated in a wide range of cancers, including colon and liver carcinomas, we investigated the impact of TOP1MT on carcinogenesis. Here, we demonstrate that lack of TOP1MT results in delayed and decreased tumor growth due to impaired mitochondrial translation. Our results reveal the importance of TOP1MT for tumor development and identify TOP1MT as a potential target for anticancer therapies.

## Results

### *TOP1MT* deficiency attenuates tumor growth in a xenograft model

**Fig. 1:**
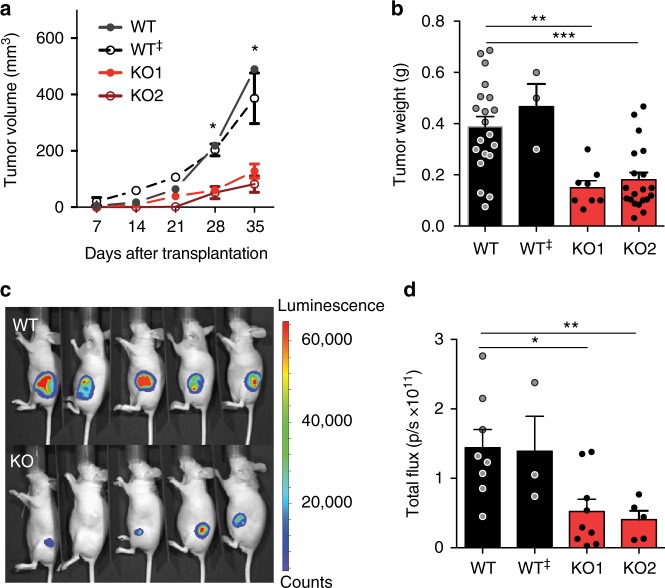
TOP1MT promotes tumor growth. **a** Tumor growth of isogenic WT and *TOP1MT* knockout HCT116 xenografts as determined by caliper measurement. Cells (10,000) from two independent *TOP1MT-*deficient clones (KO1, *n* = 5; KO2, *n* = 9), a *TOP1MT*-expressing clone (WT^‡^, *n* = 3), which went through a mock CRISPR/Cas9 process, and the parental cell line (WT, *n* = 8) were injected subcutaneously in the flanks of female Ncr-nu/nu mice. **b** Weights of excised tumors were determined after 35 days (WT, KO2, *n* = 20; WT^‡^, *n* = 3; KO1, *n* = 8). **c** Representative bioluminescence imaging 35 days after transplantation of 10,000 cells of each type. **d** Quantification of the bioluminescence imaging. The total flux is plotted as photons per second (WT, *n* = 8; WT^‡^, *n* = 3; KO1, *n* = 9; KO2, *n* = 5). All data are means ± SEM; *p < 0.05, **p < 0.01, ***p < 0.001, unpaired, two-tailed Student’s *t*-test

Based on the marked overexpression of *TOP1MT* in colon tumors (Supplementary Fig. 1a, b), we utilized HCT116 colon carcinoma cells as a model system, as this cell line shows the highest *TOP1MT* expression among the NCI-60 colon cancer cell lines. To study the function of TOP1MT in tumor development, we transplanted *TOP1MT*-deficient and *TOP1MT*-proficient HCT116 colon carcinoma cells generated by CRISPR/Cas9 (Supplementary Fig. 1c) into the flank of female nude mice^20^. *TOP1MT*-KO cells shared the same morphology and cell size compared with the parental cell line (Supplementary Fig. 1d). Lack of *TOP1MT* significantly attenuated tumor growth (Fig. 1a) in two independent *TOP1MT* knockout clones (KO1, *p* = 0.0004; KO2, *p* = 0.007, *t*-test), whilst no difference in tumor growth was observed between the parental cells and *TOP1MT*-expressing cells that underwent parallel genomic editing procedure (WT^‡^). Tumor weight after dissection was significantly lower in the *TOP1MT*-KO tumors compared to WT tumors (Fig. 1b; KO1: *p* = 0.001, KO2: *p* = 0.0001, *t*-test). Additionally, bioluminescence imaging reflected the reduction of tumor growth in the absence of *TOP1MT* (Fig. 1c, d; KO1: *p* = 0.009, KO2: *p* = 0.01, *t*-test). Transplantation of a mix of WT and *TOP1MT*-KO cells (Supplementary Fig. 1e) also showed that the arising tumors were mostly formed by *TOP1MT* WT cells (Supplementary Fig. 1f).

To elucidate the impact of *TOP1MT* on tumor formation, we performed limiting dilution assay^22^. Lack of *TOP1MT* decreased the frequency of tumor-initiating cells over 20-fold (from 1/1608 to 1/72 when compared to the parental cell line; Table 1), suggesting that *TOP1MT* impacts the tumor-initiating cell potential. Overall, we could not detect any difference in tumor-initiating frequency, growth kinetics or weight between WT and control WT^‡^ derived tumors, excluding potential off-target effects of the CRISPR/Cas9 process. These results provide the first evidence that *TOP1MT* promotes tumor growth.

**Table 1:** Limiting dilution analyses

Cell line	Weeks	Tumor incidence/injected cells			TIF	CI 95%	*P*-value
		100	1000	10,000			
WT	3	3/4	4/4	4/4	1/72	1/21–1/245	
WT^‡^	3	2/4	4/4	4/4	1/142	1/37–1/548	ns
TOP1MT KO	3	0/4	2/4	4/4	1/1608	1/447–1/5787	<0.001

The frequency of tumor-initiating cells (TIF) and confidence interval (CI 95%) were calculated based on the number of resulting tumors per injection site after 3 weeks (*n* = 4)

ns, not significant

### *TOP1MT* diminishes dependency of tumor cells on glucose

**Fig. 2:**
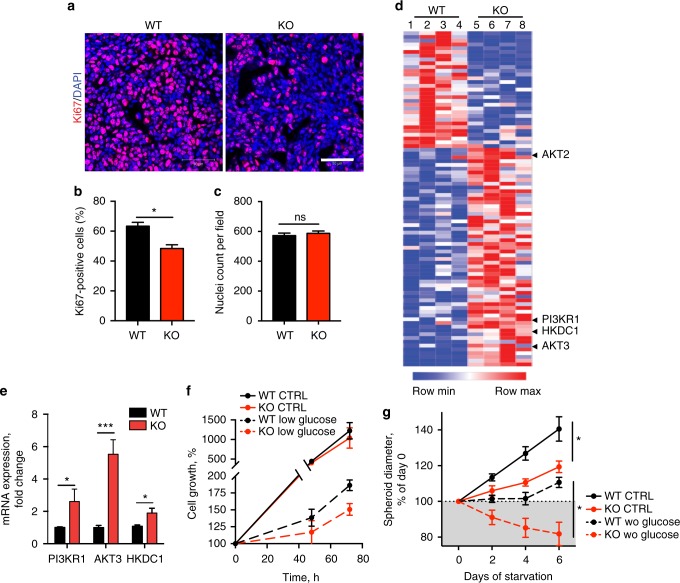
Knocking out *TOP1MT* restrains cell proliferation and sensitizes cells to glucose starvation. **a** Representative Ki67 immunofluorescence staining of WT and *TOP1MT*-KO xenograft tumors. Scale bar, 50 μm. **b**, **c** Quantification of the fraction of Ki67-positive cells (**b**) and nuclei count per field (**c**) measured by ZEN software (6 images per animal, 5 animals per genotype). **d** Heat map showing significant changes in gene expression profiles analyzed with the nCounter PanCancer Progression panel in four *TOP1MT*-deficient vs. four WT tumors (89 genes, *p* < 0.05). **e** Transcript levels of selected genes of the PI3K/AKT pathway determined by RT-qPCR (*n* = 4, each performed in triplicates). **f** Kinetics of cell growth under standard culture conditions and under glucose withdrawal (1 g L^−1^ glucose, dashed lines) (four independent experiments performed in quadruplets). **g** Growth of WT and *TOP1MT*-KO multicellular tumor spheroids (MCTS) formed by 10,000 HCT116 cells in six independent experiments, each performed in quintuplets. Day 0 corresponds to 48 h after cell seeding (spheroid maturation). Dashed and solid lines represent growth in the absence and presence of glucose, respectively. Data represent the means ± SEM, *p < 0.05, **p < 0.01, ***p < 0.001, Student’s *t*-test

Next, we tested whether the reduced growth of *TOP1MT*-KO tumors was due to decreased cell proliferation. Accordingly, Ki67 staining showed that tumors arising from *TOP1MT*-deficient cells were significantly less proliferative compared to WT cells (Fig. 2a, b; *p* = 0.026, *t*-test), while no difference in nuclei count per field was observed excluding potential cell size alterations (Fig. 2c, *p* = 0.53, *t*-test). Additionally, apoptosis was enhanced in the *TOP1MT*-deficient xenograft tumors (Supplementary Fig. 2a, b). However, overall apoptosis was low, suggesting that decreased proliferation is the main determinant for the slower growth rate of tumors derived from *TOP1MT*-KO cells.

To elucidate the underlying transcriptional differences between *TOP1MT*-KO and WT tumors, we analyzed gene expression using the nCounter PanCancer Progression panel. Lack of *TOP1MT* resulted in the activation of the phosphoinositide 3-kinase PI3K/AKT signaling pathway (Fig. 2d, Supplementary Data 1 and Supplementary Table 1). Upregulation of the key enzymes *AKT3, PI3KR1* and *HKDC1* was confirmed by RT-qPCR and western blotting (Fig. 2e, Supplementary Fig. 2c, *PI3KR1*: *p* = 0.04; *AKT3*: *p* = 0.0001; *HK*: *p* = 0.04, *t*-test). As AKT activation exerts a direct influence on cellular glucose homeostasis^23^, we tested gene expression of the two key regulators of glycolysis, 6-phosphofructo-2-kinase/fructose-2,6-biphosphatase 1 (*PFKFB1)* and hexokinase domain containing 1 (*HKDC1)*. Both were upregulated 2- to 3-fold in the *TOP1MT-*KO tumors measured by the nCounter PanCancer Progression panel. These findings indicate that loss of *TOP1MT* is associated with activation of the PI3K/AKT pathway, potentially increasing glucose utilization.

To test this possibility, we then determined growth of HCT116 cells in the presence or absence of TOP1MT under glucose restriction (Fig. 2f). Under standard cell culture conditions, HCT116 WT and *TOP1MT*-KO cells grew indistinguishably. However, *TOP1MT*-KO cells were more sensitive to glucose withdrawal than WT cells (Fig. 2f). We hypothesize that lack of *TOP1MT* can be compensated by the presence of other topoisomerases under basal growth conditions^18^, while this redundancy becomes restricted in a microenvironment where supply of nutrients, oxygen, signaling molecules, and metabolites is limited. Accordingly, we observed impaired growth of HCT116 *TOP1MT*-KO cells under serum starvation (Supplementary Fig. 2d, *p* = 0.01, multiple *t*-tests with correction for multiple comparisons using the Holm-Sidak method). Further, murine embryonic fibroblasts (MEF) lacking TOP1MT were more sensitive to a nutrient and oxygen restricted environment than WT cells (Supplementary Fig. 2e, f; *p* = 0.0001, *p* = 0.007, multiple *t*-tests with correction for multiple comparisons using the Holm-Sidak method). As 3D cultures mimic more closely an *in vivo* tumor microenvironment by creating a gradient of nutrients, oxygen, and catabolites^24^, we determined the impact of TOP1MT on the growth of multicellular tumor spheroids (MCTS). Forty-eight hours after seeding, cells of both genotypes formed similarly sized spheroids indicating that lack of TOP1MT did not affect spheroid maturation (Supplementary Fig. 2g, *p* = 0.7, *t*-test). However, MCTS grew significantly slower in the absence of TOP1MT compared to the WT spheroids after maturation (Fig. 2g, *p* = 0.0004, multiple *t*-tests with correction for multiple comparisons using the Holm-Sidak method), reflecting the dependency of *TOP1MT*-KO cells on sufficient nutrient and oxygen supply. Similarly, glucose withdrawal resulted in a slow but steady growth of WT spheroids, whereas *TOP1MT*-KO spheroids failed to increase in size at any given time point.

These results along with the concomitant activation of the PI3K/AKT pathway prompted us to investigate the glycolytic activity in primary tumor cells isolated from the WT and *TOP1MT*-KO xenografts. We found that the glycolytic rate, as measured by the extracellular acidification rate (ECAR), remained the same in the absence or presence of *TOP1MT* (Supplementary Fig. 2h), suggesting that cancer cells already operate at their maximum glycolytic capacity. The inability to utilize other fuels to maintain proliferation in *TOP1MT*-KO cells implies impaired mitochondrial function, which is consistent with previous work in *Top1mt*-KO murine embryonic fibroblasts (MEF)^19^.

### Mitochondria are dysfunctional in *TOP1MT*-deficient tumors

**Fig. 3:**
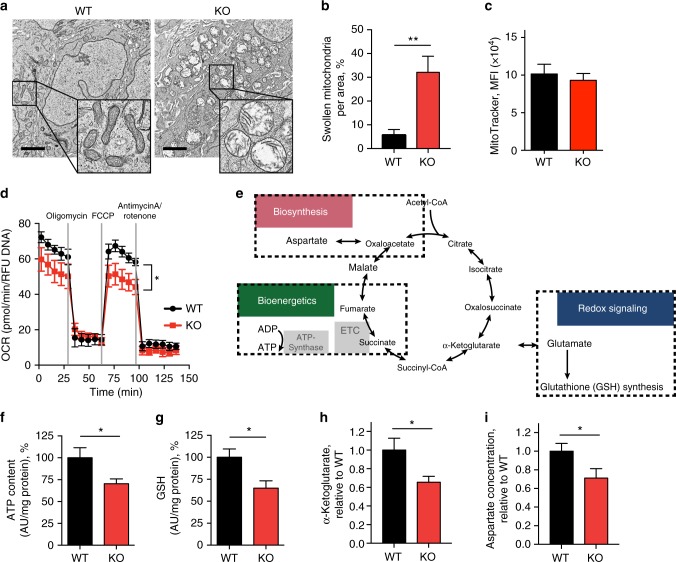
Mitochondria are defective in *TOP1MT*-KO HCT116 tumor xenografts. **a** Representative electron micrographs. The insets depict higher magnification of the boxed areas. Scale bar, 2 μm. **b** Quantification of swollen mitochondria in WT and *TOP1MT*-KO tumors. At least 48 images were analyzed from 12 independent areas at ×5000 magnification. **c** Mitochondrial mass determined by MitoTracker Deep Red FM staining of tumor cells isolated from WT and *TOP1MT*-KO tumor xenografts. The median ± SEM is plotted (*n* = 5 for each genotype). **d** Differential oxygen consumption rate measured by Seahorse XF96 Extracellular Flux Analyzer in WT and *TOP1MT*-KO cells isolated from tumor xenografts (*n* = 5 for each genotype, each performed in quintuplets). **e** Scheme of mitochondrial functions for cellular biosynthesis, bioenergetics and redox signaling. **f** Cellular energy levels measured by ATPlite (*n* = 7). **g** Redox state determined by glutathione levels (*n* = 5). **h** Steady-state level of the TCA cycle metabolite α-ketoglutarate, *n* = 10-12. **i** Reduced aspartate levels in *TOP1MT*-KO tumor xenografts (*n* = 10). Data represent mean ± SEM except in **c**, *p < 0.05, **p < 0.01, Student’s *t*-test

To elucidate how *TOP1MT* affects mitochondria in tumor cells, we analyzed the *TOP1MT*-KO and WT xenograft tumors by electron microscopy. It revealed the presence of a substantial fraction of swollen mitochondria in *TOP1MT*-KO tumors (Fig. 3a, Supplementary Fig. 3a, b) characterized by a lucent-swelling matrix and cristae disarrangement or partial cristolysis (Fig. 3a, inset). As the enzymes involved in oxidative phosphorylation are located on the inner mitochondrial membrane, mitochondrial swelling with distortion of cristae has been associated with the incapability to produce adequate amounts of ATP by mitochondrial respiration^25^. Quantification showed that the frequency of swollen mitochondria increased over 5-fold in *TOP1MT*-KO tumors compared to WT (Fig. 3b, *p* = 0.001, *t*-test), while no alteration in the average mitochondrial content was observed in the absence of TOP1MT (Fig. 3c, Supplementary Fig. 3c, *p* = 0.6, *t*-test). Extracellular flux analysis also showed the mitochondrial dysfunction of primary *TOP1MT*-KO tumor cells with reduced respiratory activity and significantly decreased maximal respiratory capacity (Fig. 3d, Supplementary Fig. 3d, *p* = 0.04, *t*-test).

Mitochondria represent not only important hubs for cellular metabolism but also function as signaling platforms^26^. In particular, they play a central role in redox homeostasis, catabolic processes producing bioenergy, as well as anabolic processes supplying macromolecules for cellular proliferation (Fig. 3e). Hence, we tested whether the morphological alterations and reduced respiratory rate in the *TOP1MT*-KO tumor cells might affect the biochemical functions of mitochondria. Accordingly, *TOP1MT*-deficient tumors showed significantly reduced ATP levels (Fig. 3f, *p* = 0.039, *t*-test) and enhanced oxidative stress, as determined by glutathione levels (Fig. 3g, *p* = 0.02, *t*-test). Additionally, loss of *TOP1MT* was associated with perturbations in the electron transport chain measured by a significant decrease in the tricarboxylic acid cycle (TCA) metabolite α-ketoglutarate, which after metabolic conversion to glutamate serves as precursor for glutathione (Fig. 3h, *p* = 0.02, *t*-test). Aspartate has been reported to be essential for cellular proliferation by ensuring sufficient supply of building blocks. As a result of impairment of the electron transport chain aspartate levels drop^27,28^. Consistently, aspartate levels were significantly diminished in *TOP1MT*-deficient tumors (Fig. 3i, *p* = 0.04, *t*-test). Thus, we conclude that mitochondrial dysfunction caused by the loss of *TOP1MT* induces oxidative stress, reduces energy supply and impairs the anabolic function of mitochondria limiting building blocks, ultimately resulting in suppressed tumor growth.

### *TOP1MT* deficiency impairs mitochondrial translation

**Fig. 4:**
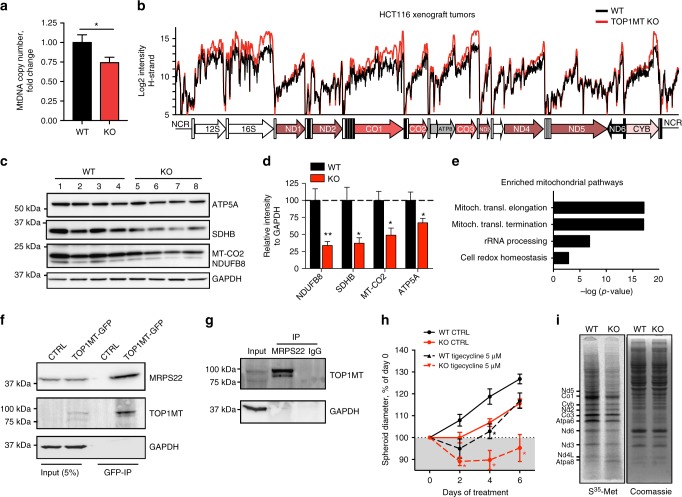
Lack of *TOP1MT* impairs mitochondrial translation. **a** Reduced mtDNA copy number was determined by RT-qPCR in *TOP1MT*-KO and WT HCT116 tumor xenografts (*n* = 9, each genotype, each performed in triplicates). **b** Conserved mitochondrial transcription profiles of *TOP1MT*-KO vs. WT tumor xenografts determined by tiling array^31^ (*n* = 4, each genotype). **c** Representative western blots showing reduced levels of mitochondrial OXPHOS proteins in *TOP1MT*-KO tumor xenografts (lane 5–8). **d** Quantification of mitochondrial OXPHOS proteins (*n* = 7, each genotype). **e** Gene ontology analysis of TOP1MT binding partners identified by TOP1MT immunoprecipitation followed by mass spectrometry. **f**, **g** Co-immunoprecipitation of TOP1MT-GFP (**f**) and MRPS22 (**g**) followed by western blotting. **h** Reduced growth of *TOP1MT*-KO HCT116 multicellular tumor spheroids. Day 0 corresponds to 48 h after cell seeding (spheroid maturation); *n* = 5, each performed in quintuplets. Dashed and solid lines represent spheroids treated with and without (CTRL) 5 μM tigecycline (*n* = 5, each performed in quintuplets), respectively. **i** Reduced mitochondrial protein synthesis measured by [^35^S]-methionine labeling of WT and *Top1mt*-KO MEFs. A representative gel shows the autoradiography of newly synthesized mitochondrial proteins (left). Equal protein loading was ensured by Coomassie staining (right). Data are mean ± SEM; *p < 0.05, **p < 0.01, Student’s *t*-test

To examine the molecular mechanism underpinning the mitochondrial dysfunction of *TOP1MT*-KO cancer cells, and because TOP1MT is known to act primarily as a DNA processing enzyme^17,18,29^, we examined mtDNA copy number and transcripts. Consistent with our prior studies in normal tissues from *Top1mt*-KO mice^18–20,30^, mtDNA copy number was decreased in the *TOP1MT*-deficient xenograft tumors compared to WT tumors (Fig. 4a, *p* = 0.04, *t*-test). Sequencing of mtDNA revealed very few significant shifts in mtDNA heteroplasmy, with more frequent alternations occurring in the non-coding regulatory regions (Supplementary Fig. 4a). Notably, in spite of decreased mtDNA, mitochondrial transcript levels were increased in *TOP1MT*-KO tumor cells per mtDNA molecule (Fig. 4b, Supplementary Fig. 4b), which is consistent with our recent findings in *Top1mt*-KO MEFs^31^. Although mitochondrial transcripts were elevated, steady-state levels of the respiratory chain proteins, encoded by both mitochondrial and nuclear DNA, were significantly diminished in *TOP1MT*-KO tumors (Fig. 4c, d, NDUFB8: *p* = 0.003, SDHB: *p* = 0.01, CO2: *p* = 0.01, ATP5A: *p* = 0.04, *t*-test).

Likewise, a reduction of complex IV was observed by immunofluorescence staining for the subunit MT-CO2 in the *TOP1MT*-KO xenograft sections (Supplementary Fig. 4c, d). It has been previously reported that defective mitochondrial protein synthesis is often associated with increased transcript levels as part of a compensatory mechanism to overcome impaired oxidative phosphorylation^32,33^. Thus, our results reveal a function of *TOP1MT* in mitochondrial translation in addition to its roles in the release of DNA torsional stress^18,29^ and mitochondrial transcription^31^.

To gain further evidence for the role of TOP1MT in mitochondrial translation and to identify potential binding partners of TOP1MT, we performed pulldown experiments of TOP1MT followed by mass spectrometry. Nearly half of the detected proteins were involved in mitochondrial translation and constituents of the mitoribosome (Supplementary Data 2). The association of TOP1MT with mitochondrial translation was also reflected in gene ontology enrichment analysis, showing significant scores for processes associated with mitochondrial translation, rRNA processing, and cell redox homeostasis (Fig. 4e). Binding of TOP1MT to MRPS22, the small subunit of the mitoribosome was further established by co-immunoprecipitation experiments of TOP1MT-GFP or MRPS22 (Fig. 4f and g). To test the functional impact of TOP1MT on mitochondrial translation, we challenged *TOP1MT*-KO and WT MCTS with tigecycline, a mitochondrial translation inhibitor^34^. *TOP1MT*-deficient spheres displayed increased sensitivity to tigecycline compared to their WT counterparts indicating altered mitochondrial translation in *TOP1MT*-KO cells (Fig. 4h, *p* = 0.032, *t*-test). To further corroborate a functional role of TOP1MT in mitochondrial translation, we labeled newly synthesized mitochondrial proteins with [^35^S]-methionine in the presence of emetine, which selectively inhibits cytoplasmic translation. Mitochondrial translation was decreased in *Top1mt*-deficient MEFs compared to WT cells (Fig. 4i). HCT116 cells lacking *TOP1MT* showed only a mild reduction of mitochondrial translation under standard culture conditions (Supplementary Fig. 4e). However, pretreatment with tigecycline followed by a short release period markedly impaired mitochondrial translation in *TOP1MT*-KO compared to WT cells indicating the role of TOP1MT in efficient mitochondrial translation both in human and murine cells. Taken together, these findings indicate a novel role of TOP1MT in mitochondrial translation and its association with mitoribosomes.

### Lack of *TOP1MT* suppresses hepatocarcinogenesis

**Fig. 5:**
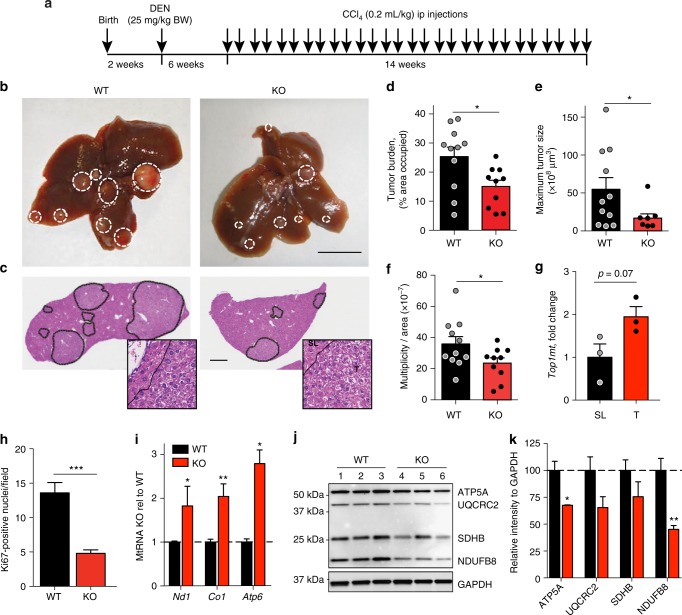
TOP1MT promotes tumor growth in a mouse model of liver carcinogenesis. **a** Experimental design. Male mice received a single intraperitoneal (i.p.) administration of diethylnitrosamine (DEN, 25 mg kg^−1^ body weight) 14 days after birth. Beginning at 8 weeks, mice received biweekly injections of carbon tetrachloride (CCl_4_, 0.2 mL kg^−1^) for 14 weeks. **b** Representative images of liver tumors in WT and *Top1mt−/−* livers at the end of treatment. Tumors are encircled with white dashed circles. Scale bar, 1 cm. **c** Hematoxylin and eosin (H&E) staining of representative paraffin-embedded liver sections. Tumor areas are encircled with black dashed lines. Scale bar, 1 mm. **d** Quantification of tumor burden expressed as proportion of hepatic parenchyma occupied by tumor tissue on H&E sections, *n* = 10-11. **e**, **f** Maximum tumor size (**e**) and tumor number (**f**) determined by analysis of H&E sections, *n* = 10-11. **g** Transcript levels of *Top1mt* in surrounding liver versus tumor tissue (*n* = 3, each performed in duplicates). **h** Quantification of Ki67-positive cells in the liver tumors (*n* = 5). **i** Transcript levels of selected mitochondrial-encoded genes in *Top1mt−/−* liver tumors relative to WT tumors (*n* = 3) determined by mitochondrial tiling array. **j** Representative western blots depicting protein levels of mitochondrial OXPHOS proteins in WT (lane 1–3) and *Top1mt-/-* (lane 4-6) tumors. GAPDH was used as loading control. **k** Quantification of mitochondrial OXHOS protein levels in WT and *Top1mt-/-* liver tumors relative to GAPDH (*n* = 3). Data are presented as mean ± SEM. *p < 0.05, **p < 0.01, ***p < 0.001, Student’s *t*-test

Next, we extended our findings on the tumor-promoting role of *TOP1MT* in human colon cancer cell xenografts to another cancer model. Given the fact that *TOP1MT* is frequently overexpressed in human hepatocellular carcinomas (HCC) (Supplementary Fig. 1a, b), and based on our previous work demonstrating that TOP1MT enhances liver regeneration^20^, we utilized our *Top1mt*-KO mouse line^18^ to induce liver cancer. Due to a low propensity of C57BL/6 mice to develop HCC, male *Top1mt*−/− and WT (*Top1mt+/+*) littermates were subjected to a combined treatment with the mutagen diethylnitrosamine (DEN) and the hepatotoxin carbon tetrachloride (CCl_4_). This model shares features with human liver cancer, which predominantly develops in the background of chronic liver inflammation caused by hepatitis B or C viruses, alcoholic steatohepatitis, or nonalcoholic steatohepatitis^35^. After 22 weeks of treatment with DEN/CCl_4_ (Fig. 5a), all WT and *Top1mt*^−/−^ mice developed liver tumors (Fig. 5b). Histologically, DEN/CCl_4_-induced tumors were indistinguishable (Fig. 5c, Supplementary Fig. 5a). However, *Top1mt*−/− mice showed reduced tumor burden (~40%) (Fig. 5d, *p* = 0.019, *t*-test) due to a significant decrease of tumor number and size (Fig. 5e, f; *p* = 0.04 and *p* = 0.03, *t*-test).

Consistent with the findings in human HCC (Supplementary Fig. 1a–b), *Top1mt* gene expression levels were upregulated 1.9-fold in murine HCC as compared to surrounding liver (Fig. 5g, *p* = 0.07, *t*-test). To exclude a contribution of hepatic fibrosis caused by carbon tetrachloride treatment to the observed differences in hepatocarcinogenesis, we stained liver tissue sections for collagen using a Picro Sirius Red staining (Supplementary Fig. 5a, bottom). We found no obvious differences in the extent of hepatic fibrosis between WT and *Top1mt*-KO mice. Additionally, expression levels of inflammatory (*Infg*) and fibrotic (*Tgfb1*) genes were not significantly altered (Supplementary Fig. 5b–c, *p* = 0.9, *p* = 0.4, *t*-test). Furthermore, we followed tumor burden in mice injected with DEN only. Hepatic tumor burden was significantly decreased in *Top1mt*-KO mice as compared to the WT littermates at 50 weeks after DEN injection (Supplementary Fig. 5d, *p* = 0.04, *t*-test). Thus, loss of TOP1MT rather than the secondary effects triggered by CCl_4_ treatment contributes to the suppression of tumor burden in *Top1mt*-KO mice.

In line with the results of the HCT116 xenograft study, *Top1mt*-deficient liver tumors were less proliferative (Fig. 5h, Supplementary Fig. 5a, *p* = 0.000001, *t*-test) and displayed activation of glucose uptake (Supplementary Fig. 5e, *p* = 0.04, *p* = 0.048, *t*-test). Mitochondrial transcript levels were also significantly increased in the liver tumors of *Top1mt*−/− mice compared to the WT counterparts, while no difference was observed in mitochondrial transcript levels in the non-cancerous livers from *Top1mt*−/− and WT mice (Fig. 5i, Supplementary Fig. 5f, *Nd1*: *p* = 0.01, *Co1*: *p* = 0.001, *Atp6*: *p* = 0.04, *t*-test). Protein levels of the OXPHOS enzyme subunits were significantly decreased in the *Top1mt*−/− liver tumors consistent with our findings in the xenograft model (Fig. 5j, k; ATP5A: *p* = 0.02, NDUFB8: *p* = 0.009, *t*-test). These results further establish the tumor-promoting role of TOP1MT and its role in enforcing mitochondrial translation.

### Relevance of TOP1MT expression signature for HCC patients

**Fig. 6:**
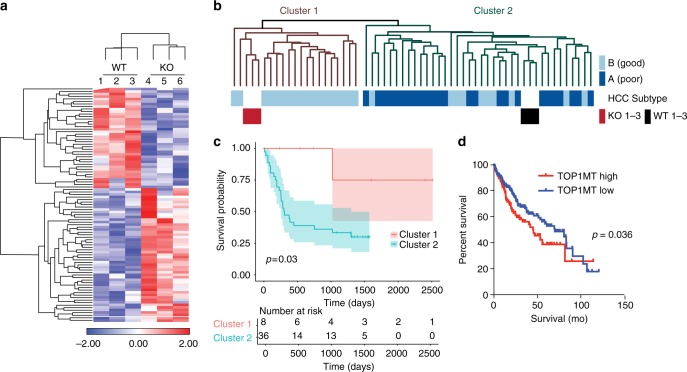
*TOP1MT*-KO gene expression signature and expression predict survival of HCC patients. **a** Supervised hierarchical clustering of gene expression profiles from 3 independent *Top1mt*-KO and WT mouse HCCs. **b** Integrative cluster analysis of murine HCCs applied to 53 human HCC patient samples using orthologous genes. Light blue bars indicate HCC patients with good survival prognosis, dark blue bars, HCC patients with poor survival, dark red bars, murine *Top1mt*-KO HCC, and black bars, murine HCC expressing *Top1mt*. **c** Overall survival of HCC patients based on the *TOP1MT* gene expression signature. **d** High expression of *TOP1MT* is associated with poor survival of patients with HCC (370 total cases from the TCGA database including 138 patients with high *TOP1MT* expression and 232 patients with low *TOP1MT* expression)

To explore the potential clinical relevance of a *Top1mt* gene expression signature, we first performed transcriptome analysis of WT and *Top1mt*-deficient tumors of similar size. Supervised hierarchical clustering of 93 differentially expressed genes (Supplementary Data 3) separated the murine liver tumors into two clusters depending on the *Top1mt* genotype (Fig. 6a). Pathway analysis of the differentially expressed mitochondrial genes showed enrichment of genes related to folate, glycine, serine, threonine, and carbon metabolism (Supplementary Fig. 6a, b), and molecular function analysis revealed a link with ribosomal function (Supplementary Fig. 6c). Additionally, PERK together with ATF4 were identified as upstream regulators (Supplementary Fig. 6d), which both have been reported as key players in the mitonuclear stress response by reducing total protein synthesis^36^. Taken together, these data further support the role of TOP1MT in mitochondrial translation.

Next, we applied the *Top1mt* gene signature derived from the murine liver tumors to a cohort of 53 HCC patients^37^. The HCC data set was obtained from Caucasian and Chinese patients using Illumina bead chips. Integrative cluster analysis of orthologous genes led to the separation of the HCC samples into two clusters (Fig. 6b). Notably, the *Top1mt*-deficient liver tumors clustered with the patients with good prognosis, while the more aggressive WT liver tumors clustered with patients characterized by a poor survival (Fig. 6b, c). This finding is in line with TCGA data showing a reverse relationship between the *TOP1MT* expression levels and prognosis for HCC patients (Fig. 6d, *p* = 0.036). To mitigate concerns that proliferation and cell cycle-related genes significantly contribute to the observed correlation between *TOP1MT* expression and prognosis, we computed the overlap between our *Top1mt*-KO gene expression signature and a curated consensus catalog of cell cycle regulated genes^38^. The two signatures did not overlap supporting our conclusion that the *Top1mt*-KO signature is not driven by the differences in the proliferation state (Supplementary Fig. 6e).

Finally, we addressed the question whether the observed correlation between gene expression and prognosis was a common property of type I topoisomerases in the mitochondria or whether it was specific for TOP1MT. Notably, TOP3α is upregulated only in a very small fraction of HCC patients (4.8% versus 37% patients with high *TOP1MT* expression) and *TOP3α* expression levels did not correlate with patient survival. Likewise, upregulation of *TOP3β* was found only in 22 out of 371 patients (5.9%) and did not correlate with survival (Supplementary Fig. 6f, g). Thus, we conclude that the correlation between good clinical prognosis and low *TOP1MT* gene expression is specific for TOP1MT. Overall, these findings demonstrate the role of TOP1MT in promoting tumor growth and support the prognostic value of the *TOP1MT* genomic signature for HCC patients.

## Discussion

Mitochondria are emerging as essential organelles for the survival and proliferation of cancer cells, as well as potential targets for cancer therapy^39,40^. Here, we introduce the role of *TOP1MT*, the only topoisomerase exclusively localized to mitochondria^10,17^, in carcinogenesis.

Based on two independent in vivo tumor models, we show that TOP1MT promotes tumor growth by enabling sustained cancer cell proliferation in an environment in which oxygen and nutrient supplies are limited. TOP1MT has been shown to play a vital role in mtDNA homeostasis by limiting mtDNA negative supercoiling^18^ and ensuring efficient mtDNA replication^20^, which becomes critical in the absence of TOP3α^41^. Thus, reduced mtDNA expansion might at least partially account for the observed decrease in cancer cell proliferation in the absence of TOP1MT. Conversely, high *TOP1MT* expression levels are commonly observed in aggressive cancers (as observed in HCCs).

An unexpected finding of the present study is that, in addition to its canonical role on mtDNA, TOP1MT directly impacts mitochondrial translation through interaction with proteins of the small mitoribosomal subunit (and specifically MRPS22). We also demonstrate that lack of TOP1MT sensitizes cells to the mitochondrial translation inhibitor tigecyline. The detailed processes involved in posttranscriptional modification of polycistronic mRNAs and the mechanism of mitoribosome assembly are not fully understood^42,43^. Early steps of mitoribosomal assembly have been shown to occur at mitochondrial nucleoids, with mitoribosomal proteins of the small subunit being more abundant in nucleoid preparations than the large subunit proteins^44^. The early intermediates of either subunit might be liberated from the nucleoids to RNA granules for further RNA processing and mitoribosome assembly due to steric reasons^45^. Mitochondrial RNA granules contain RNA-modifying enzymes including the RNase P complex and members of the FASTK family and RNA helicases, but also proteins of both the small and large mitochondrial ribosomal subunits^46^. The protein GRSF1 (G-rich sequence binding factor 1), which plays a crucial role in mitochondrial RNA processing and translational regulation, was found to localize to RNA granules^47,48^. As TOP1MT was recently found in the mitochondrial RNA granule proteome by immunoprecipitation of GRSF1^48^, we hypothesize that TOP1MT, as part of nucleoids and RNA granules, contributes to the regulation of mitochondrial translation and ribosome assembly.

Notably, mitochondrial mRNAs are overexpressed in *TOP1MT*-KO cancer cells (Fig. 4b, Supplementary Fig. 5f), which is in line with our recent genomic analyses of mitochondrial transcription^31^. The upregulation of mitochondrial transcripts could result from a compensatory mechanism in the context of impaired mitochondrial translation. Such compensation has been found in various mouse models with defective mitochondrial protein synthesis^32,33^. The precise control of mtDNA homeostasis, RNA processing, and mitochondrial translation are crucial for proficient respiratory complex activity and metabolic homeostasis^49^. We propose a pleiotropic function of TOP1MT, as a key regulatory factor enabling mtDNA replication and expansion as well as mitochondrial translation.

Mitochondria and mitochondrial translation are moving into the focus as potential targets for cancer therapy^39,50–52^. Enhanced mitochondrial biogenesis and reliance on oxidative phosphorylation of cancer cells are critical for drug selectivity and preventing off-tumor effects. For instance, K-Ras mutant tumors are sensitive to mitochondrial translation inhibitors, suggesting that a combinational therapy of BRAF and MEK inhibitors together with mitochondrial translation inhibitors might be a promising approach^34^. Thus, we anticipate that TOP1MT could be a potential target for intervention in combinational therapies against K-Ras mutant tumors, as well as in cancers with upregulated *TOP1MT* or high reliance on mitochondrial biogenesis.

HCC is the most common primary malignancy of the liver, and HCC incidence and mortality rates are steadily increasing. However, effective treatment strategies for advanced stages are lacking^53^. By applying the gene expression signature from our murine *Top1mt*-deficient HCCs, we were able to divide 53 HCC patients into two subgroups with a significantly different clinical outcome. This suggests that the *Top1mt* gene expression signature derived from our murine model could be tested further to predict high-risk patients and to adapt treatment modalities.

In summary, our results present the first evidence that loss of TOP1MT suppresses tumor growth by decreasing mitochondrial translation, thereby sensitizing tumor cells to a microenvironment with limited access to oxygen and nutrients. We also propose that the *Top1mt* gene expression signature could be utilized as prognostic tool for HCC patients and present TOP1MT as a putative mitochondrial target for adjuvant chemotherapy.

## Methods

### Cell lines and reagents

HCT116 cells were obtained from the NCI Development Therapeutics Program in 2014, and authentication of this cell line was performed prior to CRISPR/Cas9 genomic editing of *TOP1MT*. Isogenic cell lines were generated as described^20^. HCT116 cells and murine embryonic fibroblasts (MEF)^19^ were cultured in DMEM supplemented with 10% fetal bovine serum and 1% penicillin/streptomycin at 37 °C in 5% CO_2_. All experiments were performed within 25 passages from thawing, and cell lines were routinely tested for mycoplasma contamination. Antibodies were obtained from the following sources (Supplementary Table 2): abcam: OXPHOS Rodent (ab110413), OXPHOS (ab110411), Ki67 (ab16667); Cell Signaling Technologies: Akt (#4691S), p-Akt (#9271S), cleaved caspase 3 (#9661), GAPDH (#5174); Thermo Fisher Scientific: MRPS22 (#PA5-52249); MT-CO2 (#MS-1372-P1); DSHB: TOP1MT (#CPTC-TOP1-MT-3), A6 (#A6 BCM-s); Sigma-Aldrich: β-actin (#A5441), Santa Cruz: IgG (#sc-2027). Picrosirius Red (#24901-250) was purchased from Polysciences Inc; tigecycline (#PZ0021), N-Nitrosodiethylamine (DEN) (#N0756), and CCl_4_ (#289116) were purchased from Sigma-Aldrich, and olive oil (#75343) from Fluka.

### Transfection of HCT116 cells

Cells were seeded in 6-well plates and at a confluency of 70% transfected using Lipofectamine3000 (Thermo Fisher) according to the manufacturers’ protocol. The green fluorescent protein (GFP)-tagged TOP1MT vector pEGFP-TOP1MT was constructed by cloning TOP1MT cDNA into the NheI and EcoRI sites of the pEGFP-N2 vector (CLONTECH) as previously described^17^.

### Xenograft study

All procedures were performed in accordance with the guidelines of the Animal Care and Use Committee (ACUC) of the National Cancer Institute, NIH. Aliquots of 10,000 cells in DMEM were mixed 1:1 with Matrigel (Corning, # 356231), and 100 μL were subcutaneously injected into the flank of athymic Ncr-nu/nu female mice (NCI-Frederick) at 5 weeks of age. Before injection, all cell lines were tested for a panel of viruses, per NIH ACUC regulations. The cells were negative for mycoplasma and all the viruses tested. Tumor growth was followed over time by caliper measurement. Tumor volume was calculated using the standard formula ½ × *L*× *W*^2^. Bioluminescence imaging was performed 35 days post transplantation. Mice were injected i.p. with 100 μL d-luciferin (Biosynth International, #L-8200) and imaged ten minutes post-injection using the IVIS Lumina system (Xenogen, Caliper Life Sciences). Images were analyzed using the Living Image software.

### Immunocytochemistry

Xenograft and liver sections were fixed overnight at 4 °C in zinc formalin fixative (Sigma, #Z2902). Five micrometre paraffin sections were stained with Ki67 (1:100), cleaved caspase 3 (1:100) and MT-CO2 (1:200), or Picro Sirius Red according to the suppliers’ recommendations. Fluorescently stained tissues from 6 tumors (for each genotype) were viewed by a Zeiss LSM 880 microscope (Zeiss, Thornwood, NY). Images (at least 5 images for each tumor) were quantified and analyzed using the Zen 2012 software (Zeiss, Thornwood, NY). Nuclei count per field was determined using the Zen 2012 software (6 images per animal, 5 animals per genotype).

Hematoxylin and Eosin (H&E) staining for routine histology was performed by Histoserv, Inc. (Gaithersburg).

### NanoString analysis

Gene expression analysis of xenograft tumors (*n* = 4, each genotype) was performed using the nCounter Pancancer Progression panel (Nanostring Technologies, Seattle, WA) according to the manufacturers’ protocol. RNA was isolated with TRIzol^TM^ (Invitrogen, #15596026), and 100 ng of total RNA was used for nCounter hybridizations. Data were normalized to endogenous housekeeper controls.

### Quantitative PCR

RNA was extracted using TRIzol^TM^ (Thermo Fisher Scientific, #15596026) or RNeasy Mini Kit (Qiagen, #74104) and reverse transcribed using RevertAid RT Reverse Transcription Kit (Thermo Fisher Scientific, #K1691) according to the manufacturers’ instructions. Quantitative PCR (qPCR) was performed using TaqMan Universal PCR Mastermix (Thermo Fisher Scientific, #4304437) and TaqMan probes for *PI3KR1*, *AKT3*, *HK*, *Glut4*, *Top1mt*, and *Pparg* or PrimeTime Std qPCR Assay (Integrated DNA Technologies) for *Infg* and *Tgfb1*(Supplementary Table 3). Gene expression was normalized to *GAPDH* and *Gapdh* or *b2m* (Thermo Fisher Scientific).

### Quantification of mtDNA copy number

Total DNA was isolated from xenograft tumors using PureLink Genomic DNA Mini Kit (Thermo Fisher Scientific, #K182001) according to the manufacturers’ protocol. Quantitative PCR was performed in triplicates on 384-Well Reaction Plates (Applied Biosystem) after DNA quantification with Nanodrop1000. For each PCR reaction, 25 ng DNA, 5 μL Power SYBR-Green PCR Master Mix (Applied Biosystems), and 0.5 μM of each forward and reverse primer were used. β2-microglobulin (β2m) was used as standard reference. Primer sequences used: β2m F (5′-TGCTGTCTCCATGTTTGATGTATCT-3′); β2m R (5′-TCTCTGCTCCCCACCTCTAAGT-3′); ND1 F (5′-AAGTCACCCTAGCCATCATTCTAC-3′); and ND1 R (5′-GCAGGAGTAATCAGAGGTGTTCTT-3′).

### Mitochondrial DNA tiling array

Total RNA was isolated from human HCT116 xenografts (*n* = 4 for each genotype), murine livers (*n* = 3 for each genotype), and liver tumors (*n* = 3 for each genotype) using TRIzol^TM^ (Thermo Fisher Scientific, #15596026) before loading onto RNeasy Mini Kit columns (Qiagen, #74104) according to manufacturers’ instructions as described. Mitochondrial DNA tiling array was performed as previously described^31^.

### Immunoblot analyses

For detection of protein levels, 50 mg of xenograft tumor or murine liver tumor tissue were homogenized and lysed in RIPA buffer supplemented with 0.4 M NaCl and protease inhibitor cocktail (Roche Applied Science). Lysates were centrifuged at 15,000× *g* at 4 °C for 10 min and protein concentration was measured using the DC^TM^ Protein Assay kit (Bio-Rad) in the supernatant. Twenty micrograms of protein were loaded onto Novex^TM^ tris-glycine gels (Thermo Fisher Scientific). Blotted membranes were blocked with 5% milk in PBS with 0.1% Tween-20. The primary antibodies were diluted in 5% milk in PBST at 1:1000 for OXPHOS Human Cocktail, OXPHOS Rodent Cocktail, Akt, and MRPS22; 1:500 for p-Akt, 1:250 for TOP1MT and at 1:2000 for GAPDH and β-actin (Supplementary Table 2). Uncropped blots are included in Supplementary Figure 7.

### Immunoprecipitation and IP-mass spectrometry

IP samples were prepared using the GFP-Trap^®^ kit from Chromotek according to the manufacturers’ protocol. HCT116 cells overexpressing TOP1MT-GFP and TOP1MT-KO cells (1 × 10^7^) were collected and resuspended in 200 μL lysis buffer supplemented with 1 mg mL^−1^ benzonase (Sigma-Aldrich), 1 mg mL^−1^ RNase A, 2.5 mM MgCl_2_ and 1× protease inhibitor cocktail (Roche Applied Science). For MRPS22-IP or IgG-IP, 4 μg antibody was added and incubated for 1 h at 4 °C under agitation. Pierce Protein A/G magnetic beads (Thermo Fisher Scientific, #88802) for pulldown of MRPS22 and IgG and 25 μL of GFP-Trap beads for TOP1MT-GFP pulldown in HCT116 cells, respectively, were equilibrated in dilution buffer. Beads were added to the respective samples and incubated at 4 °C under constant agitation. After 1 h, beads were washed with 0.5 mL wash buffer three times and resuspended in 100 μL 2x SDS sample buffer (Thermo Fisher Scientific). IP samples were analyzed by western blotting and by mass spectrometry. GFP-pulldown was also performed in non-transfected cells as a background control.

For mass spectrometry, interacting proteins were fractionated by SDS-PAGE, and each lane cut into 10 slices. The protein bands were then in-gel digested with trypsin (Thermo Fisher Scientific) overnight at 37 °C as described^54^. The peptides were extracted following cleavage and lyophilized. The dried peptides were solubilized in 2% acetonitrile, 0.5% acetic acid, and 97.5% water for mass spectrometry analysis. They were trapped on a trapping column and separated on a 75 µm × 15 cm, 2 µm-Acclaim PepMap reverse phase column (Thermo Scientific) using an UltiMate 3000 RSLCnano HPLC (Thermo Scientific). Peptides were separated at a flow rate of 300 nL min^−1^ followed by online analysis by tandem mass spectrometry using a Thermo Orbitrap Fusion mass spectrometer. Peptides were eluted into the mass spectrometer using a linear gradient from 96% mobile phase A (0.1% formic acid in water) to 55% mobile phase B (0.1% formic acid in acetonitrile) over 30 min. Parent full-scan mass spectra were collected in the Orbitrap mass analyzer set to acquire data at 120,000 FWHM resolution; ions were then isolated in the quadrupole mass filter, fragmented within the HCD cell (HCD normalized energy 32%, stepped ± 3%), and the product ions analyzed in the ion trap. Proteome Discoverer 2.2 (Thermo) was used to search the data against human proteins from the UniProt database using SequestHT. The search was limited to tryptic peptides, with maximally two missed cleavages allowed. Cysteine carbamidomethylation was set as a fixed modification, and methionine oxidation set as a variable modification. The precursor mass tolerance was 10 ppm, and the fragment mass tolerance was 0.6 Da. The Percolator node was used to score and rank peptide matches using a 1% false discovery rate.

### Flow cytometry analysis of mitochondrial mass

Cells (300,000) were seeded in 6-well plates and allowed to grow for 24 h. Cells were then incubated with 100 nM MitoTracker Deep Red dye (Thermo Fisher) for 20 min in normal culture medium at 37 °C. After three washes with 3 mL warm PBS, cells were trypsinized and resuspended in PBS supplemented with 1% FBS and 2 mM EDTA and immediately analyzed by flow cytometry (BD LSR Fortessa). Data analysis was performed using FlowJo 10.4.2 (FlowJo LLC) as exemplified in Supplementary Figure 8.

### Measurement of intracellular ATP levels

Tumor tissue (50 mg) was homogenized, and ATP levels were determined using the ATPlite 1-step kit (PerkinElmer). Briefly, 50 μL ATPlite solution was added to 50 μL tissue homogenate in a 96-well white plate (Perkin Elmer Life Sciences, #6005680). After 5 min, luminescence was measured with an EnVision 2104 Multilabel Reader (PerkinElmer).

Cells at a density of 5000 cells per well were seeded in 96-well plates and allowed to attach for 4 h. Cells were grown for 48 h and 72 h in normal growth medium or incubation medium (1 g L^−1^ glucose and 1% FBS, respectively) under normoxic conditions and in a hypoxia incubator (2% oxygen), respectively. Chemiluminescence was measured after adding 100 μL ATPlite solution (Perkin Elmer).

### Glutathione measurement

Reactive oxygen species (ROS) production was measured quantifying reduced glutathione^51^ in xenograft tumor tissue. GSH levels were assessed in 50 mg tissue lysates using the luminescence-based GSH-Glo™ Glutathione Assay (Promega) according to the manufacturers’ protocol.

### Single metabolite measurement

Steady-state levels of aspartate and α-ketoglutarate in xenograft tumors were measured using the aspartate assay kit (Sigma-Aldrich, MAK095) and α-ketoglutarate assay kit (Sigma-Aldrich, MAK054), respectively, according to the manufacturers’ protocol. Briefly, 20 mg fresh tumor sample (*n* = 10–13 for each genotype) were homogenized in 100 μL ice-cold buffer. Samples were then centrifuged at 13,000× *g* for 10 min at 4 °C. Supernatant was deproteinized with a 10 kDa MWCO spin filter (Biovision, #1997-25). A volume of 50 μL was used for assay reaction which was performed according to manufacturers’ manual.

### Measurement of oxygen consumption rate

Xenograft tumors (*n* = 5 for each genotype) were minced and incubated with Accutase solution (Millipore Sigma, #SCR005) for 30 min under continuous shaking. The resulting cell suspension was filtered through a 70 μm mesh, centrifuged at 1300× *g* and resuspended in DMEM. Isolated primary tumor cells were seeded at a density of 20,000 cells/96-well. For OCR measurements, cells were incubated in medium supplemented with 2 mM glutamine, 10 mM glucose, and 1 mM pyruvate for 1 h, prior to the measurements using the XF Cell Mito Stress Kit (Agilent, #103015-100). For ECAR measurements (*n* = 3 for each genotype), cells were incubated with basal medium prior to injections using the Glycolysis Stress Test kit (Agilent, #103020-100). Experiments were run on a XF96 Extracellular Flux Analyzer (Seahorse Bioscience) in at least six replicates and raw data were normalized to DNA content measured using the CyQUANT Cell Proliferation Assay Kit (Thermo Fisher Scientific, #C7026).

### Multicellular tumor spheroid growth

Spheroids were generated from WT and *TOP1MT*-KO HCT116 cells using GravityTRAP^TM^ ULA plates (insphero, Perkin Elmer) according to manufacturers’ protocol. Briefly, 10,000 cells were seeded in 70 μL DMEM per well, and the plate was centrifuged for 2 min at 250 RCF. Spheroids were formed by cell aggregation and self-assembly to a 3D structure after 48 h (sphere maturation) which was considered as time 0. Spheroid growth was measured in quintuplets over time by determination of spheroid diameter under a bright field microscope and is represented as ratio over the matured spheroid (48 h after cell seeding, time 0). Growth or incubation medium was replaced every 48 h. Incubation medium was supplemented with 5 μM tigecycline to inhibit mitochondrial translation, and spheroid growth was followed over the duration of seven days.

### Transmission electron microscopy

Xenograft tumor samples (*n* = 2 for each genotype) were cut into small (1 × 1 mm^3^) cubes and fixed in a mix of 2% glutaraldehyde and 4% paraformaldehyde in 0.1 M cacodylate buffer. Samples were processed and examined in an electron microscope operated at 80 kV (Hitachi H7650, Tokyo by the Electron Microscopy Core at the National Cancer Institute (Frederick) as previously described^20^. At least 48 digital images taken at ×5000 magnification from 12 independent areas were captured by CCD camera (AMT, Danvers, MA) and used for a quantitative evaluation of mitochondrial morphology.

### Mitochondrial protein synthesis

Mitochondrial translation in WT and Top1mt-KO MEFs was assessed as described in ref. ^55^. Briefly WT and *Top1mt*-KO MEFs were washed with methionine/cysteine-free DMEM, 2 mM l-glutamine, 96 μg/mL cysteine and 5% (v/v) dialyzed FBS and incubated for 10 min in the same media at 37 °C. 100 μg/mL emetine was then added directly to the media in order to inhibit cytosolic translation; after 20 min proteins newly synthetized in the mitochondrial compartment were labeled with 100 μCi [^35^S]-methionine (Perkin Elmer) for 1 h at 37 °C. Cells were then washed three times with PBS and lysed in PBS, 0.1% *n*-dodecyl-β-d-maltoside (DDM, Sigma), 1% SDS, 1X protease inhibitor cocktail (Roche) and 50 U benzonase (Novagen). 25 μg of total protein lysates were subjected to SDS-PAGE (Novex), gels were dried and radiolabelled proteins were detected by Phosphorimager.

Mitochondrial translation analysis of HCT116 cells was performed as previously described with slight modifications^56^. HCT116 WT and *TOP1MT*-KO cells were seeded (1 Mio) in 6-well plates and allowed to attach. Cells were cultured in growth medium or in the presence of tigecyline (10 μM) for 16 h, followed by a 2-hour washout phase. Media was replaced with 2 mL DMEM lacking methionine or cysteine (Life Technologies) with 10% FBS for 30 min before adding 100 μL of a 2 mg mL^−1^ emetine solution for 15 min. Cells were labeled using 200 μCi mL^−1 35^S EasyTag (Perkin Elmer) for 45 min. After three washes with 2 mL PBS, normal growth medium was applied to the cells for 10 min. Cells were scraped in 1 mL ice-cold PBS, after two washes with PBS, and collected by centrifugation. Cell pellets were resuspended in RIPA buffer (50 mM Tris-HCl pH7.5, 150 mM NaCl, 1% NP-40, 1 mM MgCl_2_, 1× protease inhibitor mix and 1 mg mL^−1^ benzonase) and incubated on ice for 20 min. After centrifugation at 2000× *g* for 10 min, protein concentration was determined by BCA assay (Biorad). A volume of 50 μg lysate was loaded onto a 12% SDS-PAGE gel. The gel was dried at 70 °C for 1 h, before exposing it to a film at −80 °C for at least three days.

### mitoRCA-seq analysis

Mutation frequency was determined by mitoRCA-seq as described^57^. Quality check of Illumina DNA-Seq data was performed using FastQC 0.10.1. followed by trimming of data by Trimmomatic-0.35. mtDNA sequence analysis was performed using the MToolBox pipeline^58^ utilizing the computational resource of the NIH HPC Biowulf cluster. The Revised Cambridge Reference Sequence (RCRS) was used for alignment.

### RNA-Seq analysis

Total RNA was isolated from WT and *Top1mt*-KO murine liver tumors of the same size (3 × 5 mm) using TRIzol^TM^ (Thermo Fisher Scientific, #15596026). Quality check (RIN ≥ 8) was performed with the Agilent Bioanalyzer. RNA-Seq libraries were prepared using the TruSeq Stranded RNA Prep Kit (Illumina, #RS-122-2201) and sequenced on the Ilumina HiSeq2500 instrument (125 bp paired end reads). Preprocessing and quality check of Illumina RNA-Seq data was performed using FastQC 0.10.1. followed by trimming of data by Trimmomatic-0.36. Reads were aligned against the reference genome (Mm 8) using BWA with default parameters. Only uniquely mapped reads were used for subsequent analyses followed by read summarization with featureCounts (subread-1.5.0-p1)^59,60^. All data analysis was performed using R programing language and related packages. The output matrix from featureCounts was input into the Bioconductor package DESeq2 for differential expression analysis^61^. Significance testing was performed using Wald Test statistics. Functional networks were assessed using Ingenuity pathway analyses.

### Liver carcinogenesis model

*Top1mt*-KO mice were generated as described in C57BL/6 background^18^ and maintained by heterozygous breeding. Genotyping was done by PCR analysis of tail genomic DNA (gDNA) at 3 weeks of age. Mice were maintained at an American Association for the Accreditation of Laboratory Animal Care-accredited animal facility at the National Cancer Institute and housed in accordance with the procedures outlined in the Guide for the Care and Use of Laboratory Animals under an animal study proposal approved by the NCI Animal Care and Use Committee.

To induce liver cancer, 14-day-old male littermates received a single intraperitoneal injection of diethylnitrosamine (DEN, 25 mg kg^−1^ body weight). When mice reached 8 weeks of age, they received biweekly injections of carbon tetrachloride (CCl_4_; 0.2 ml kg^−1^ in olive oil) for 14 weeks^62^. Liver tissue was harvested 4 days after the last injection, i.e., after the peak of proliferation associated with CCl_4_-induced tissue injury. A second group of fourteen-day old male mice received a single intraperitoneal injection of DEN (25 mg kg^−1^ body weight) without tumor promotion with CCl_4_. Liver tissue was harvested 50 weeks after DEN injection.

### TCGA data analysis

All TCGA data were accessed, analyzed, and figures were generated using the cBio Cancer Genomics Portal. All the included data agree with the TCGA publication guidelines^63^.

### Statistical analysis

The statistical analyses were conducted using two-tailed unpaired Student *t*-test. For growth kinetics, multiple *t*-tests were performed with correction for multiple comparisons using the Holm-Sidak method. In Figs. 2b and 5f one-tailed unpaired Student *t*-test was performed. For RNA-seq data analysis, significance testing was performed using Wald Test statistics. All data are expressed as the mean ± SEM, except Fig. 3c and Supplementary Fig. 3c where the median ± SEM is plotted. Individual data points are plotted in the graph for all in vivo data. Number of technical and biological replicates is described in each figure legend. All data were analyzed using the software Graphpad Prism 7 (GraphPad Software, Inc.)

### Reporting summary

Further information on experimental design is available in the Nature Research Reporting Summary linked to this article.

## Supplementary Information

Supplementary Information

Supplementary Data 1

Supplementary Data 2

Supplementary Data 3

Description of Additional Supplementary Files

Reporting Summary

## Data Availability

The data sets for the RNA-seq are available from GEO under Accession code GSE122489. All data within the manuscript is available from the authors upon reasonable request. A Reporting Summary for this Article is available as Supplementary Information file.
