# Supplementary material for: The mitochondrial type IB topoisomerase drives mitochondrial translation and carcinogenesis
Source: Nat Commun. 2019 Jan 8;10:83. doi: 10.1038/s41467-018-07922-3 (PMC6325124; doi:10.1038/s41467-018-07922-3)
Supplement: Supplementary file 1 — Supplementary Information [file 41467_2018_7922_MOESM1_ESM.pdf]

Supplementary Information

The mitochondrial type IB topoisomerase drives mitochondrial translation and carcinogenesis

Baechler et al.

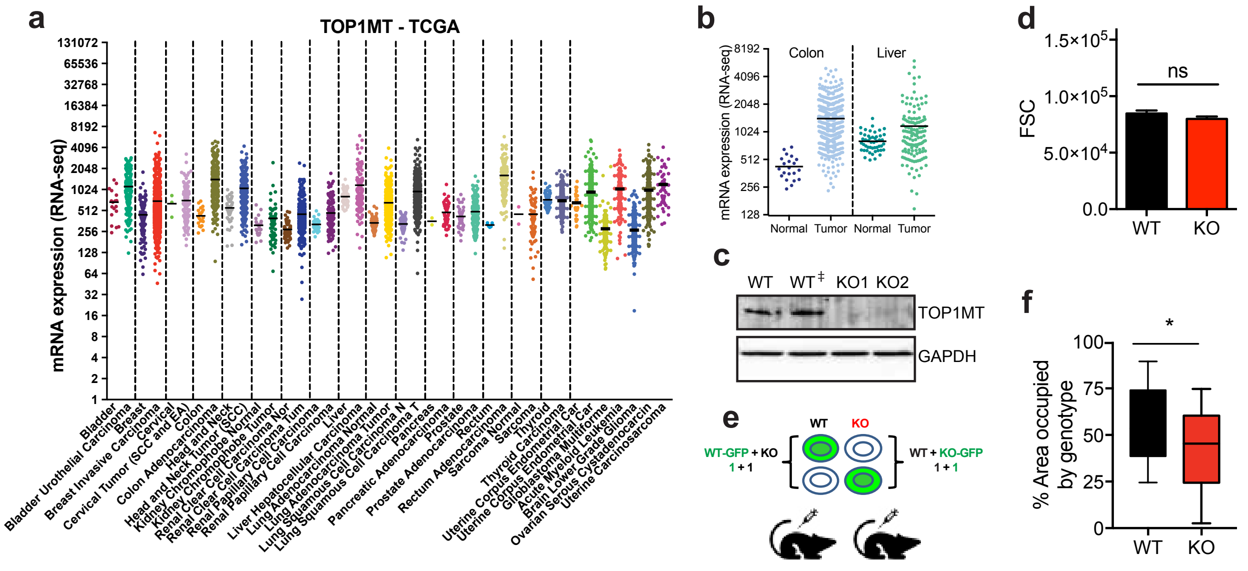

Supplementary Figure 1. The Cancer Genome Atlas (TCGA) data of *TOP1MT* expression in normal versus tumor tissues, efficiency of TOP1MT knockout in human colon cancer cell line HCT116, and effect of TOP1MT deficiency on the growth of HCT116 xenografts. **a** Comparison of *TOP1MT* expression in normal tissues versus tumor tissues according to TCGA mRNA expression data. **b** Levels of *TOP1MT* overexpression in colon and liver cancer. Enlarged view of the TCGA data. **c** Western blotting for TOP1MT in HCT116 parental cells (WT), CRISPR/Cas9-engineered cells expressing TOP1MT (WT‡), and two TOP1MT knockout clones (KO1 and KO2). GAPDH was used as a loading control. **d** Cell size of HCT116 WT and TOP1MT knockout cells analyzed by flow cytometry analysis (n=5, median ± SEM is plotted). **e** Scheme of tracking experiments using HCT116 parental (WT) and *TOP1MT* KO cells, stably transfected with green fluorescent protein (GFP). GFP-positive cells were mixed in reverse order with the corresponding unlabeled counterparts in 1:1 ratio to exclude a potential effect of GFP-toxicity on survival of WT or KO cells. A total of 10,000 cells were injected into the flank of athymic Ncr-nu/nu mice (n=3 for each combination). **f** Graph bars showing the relative contribution of WT and *TOP1MT* -KO cells to tumor growth at 35 days post transplantation. The percentage of area occupied by each genotype was determined on frozen sections using confocal microscopy by measuring GFP-positive areas versus total tumor areas. For each tumor, at least 4 images were taken at 20x magnification. All data are means ± SEM unless otherwise stated; ns, not significant; *p<0.05, Student's *t*-test.

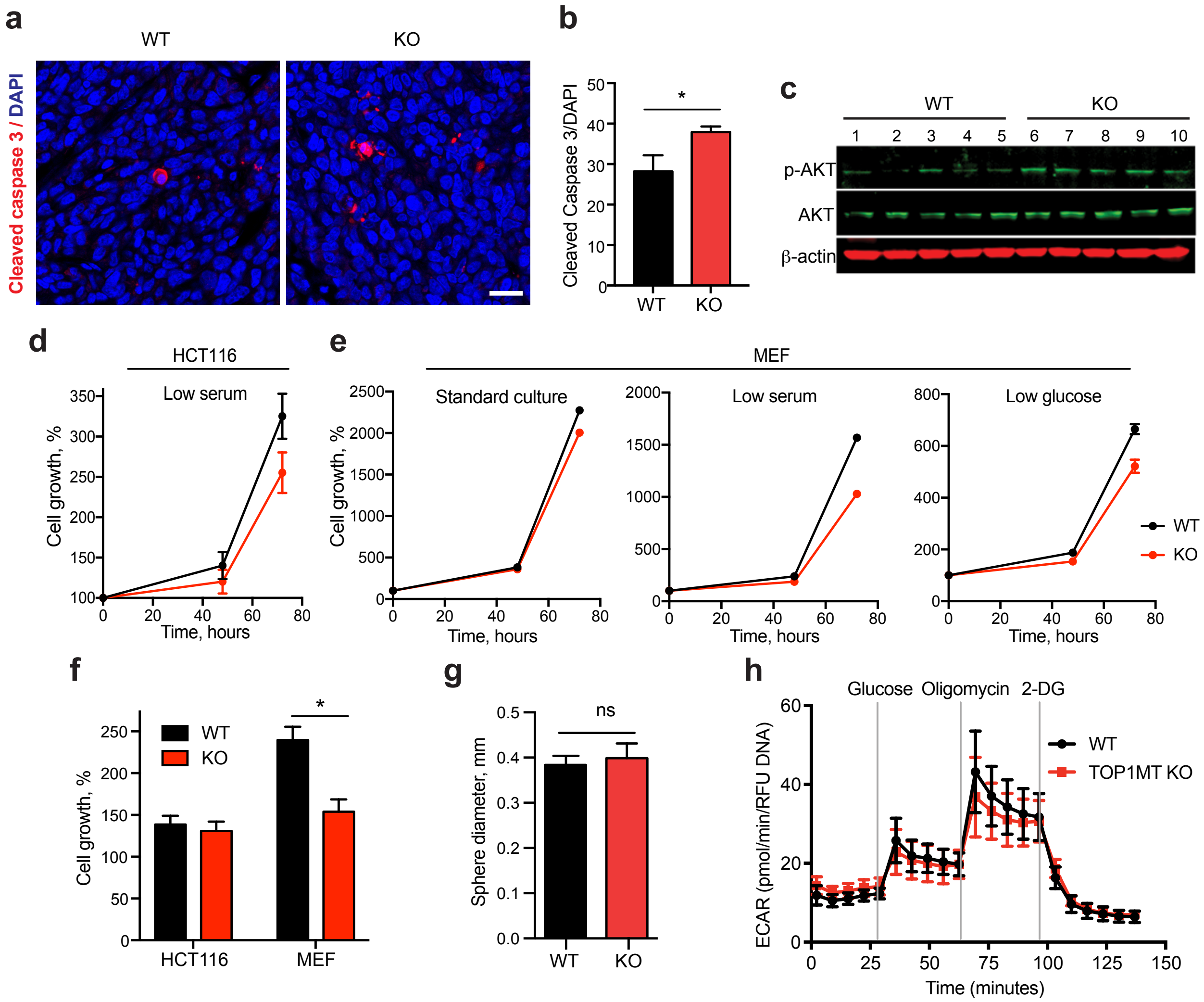

Supplementary Figure 2. Effect of TOP1MT deficiency on apoptosis, sphere formation, activation of AKT signaling, and glycolysis. **a** Representative images of immunofluorescence staining with cleaved caspase 3 in WT and *TOP1MT*-KO xenograft tumor sections. Nuclei were counterstained with DAPI. Scale bar, 20 µm. **b** Quantification of the immunofluorescence intensity of cleaved caspase 3. **c** Phosphorylation status of AKT and endogenous AKT levels in five xenograft tumors determined by Western blotting. β-actin was used as loading control. **d** Growth kinetics of HCT116 WT and *TOP1MT*-KO cells under low serum (1%) conditions measured by ATPlite 1step (Perkin Elmer; n=4 each performed in quadruplets). **e** Cell growth of murine embryonic fibroblasts (WT and *TOP1MT* KO) under nutrient restriction measured by ATPlite 1step (n=3, each performed in quadruplets). **f** Cell growth under hypoxic condition (2% oxygen) after 24 h (n=3, each performed in quadruplets). **g** Spheroid diameter 48 h after seeding of 10,000 HCT116 cells (WT and *TOP1MT* KO) in GravityTRAP ultra low attachment plates (Perkin Elmer). **h** Extracellular acidification rate (ECAR) was measured using the Seahorse BioFlux Analyzer in isolated primary WT and *TOP1MT* KO tumor cells after normalization to DNA content determined by CyQUANT (Molecular Probes, Invitrogen). All data are means ± SEM; ns, not significant; *p<0.05, Student's *t*-test.

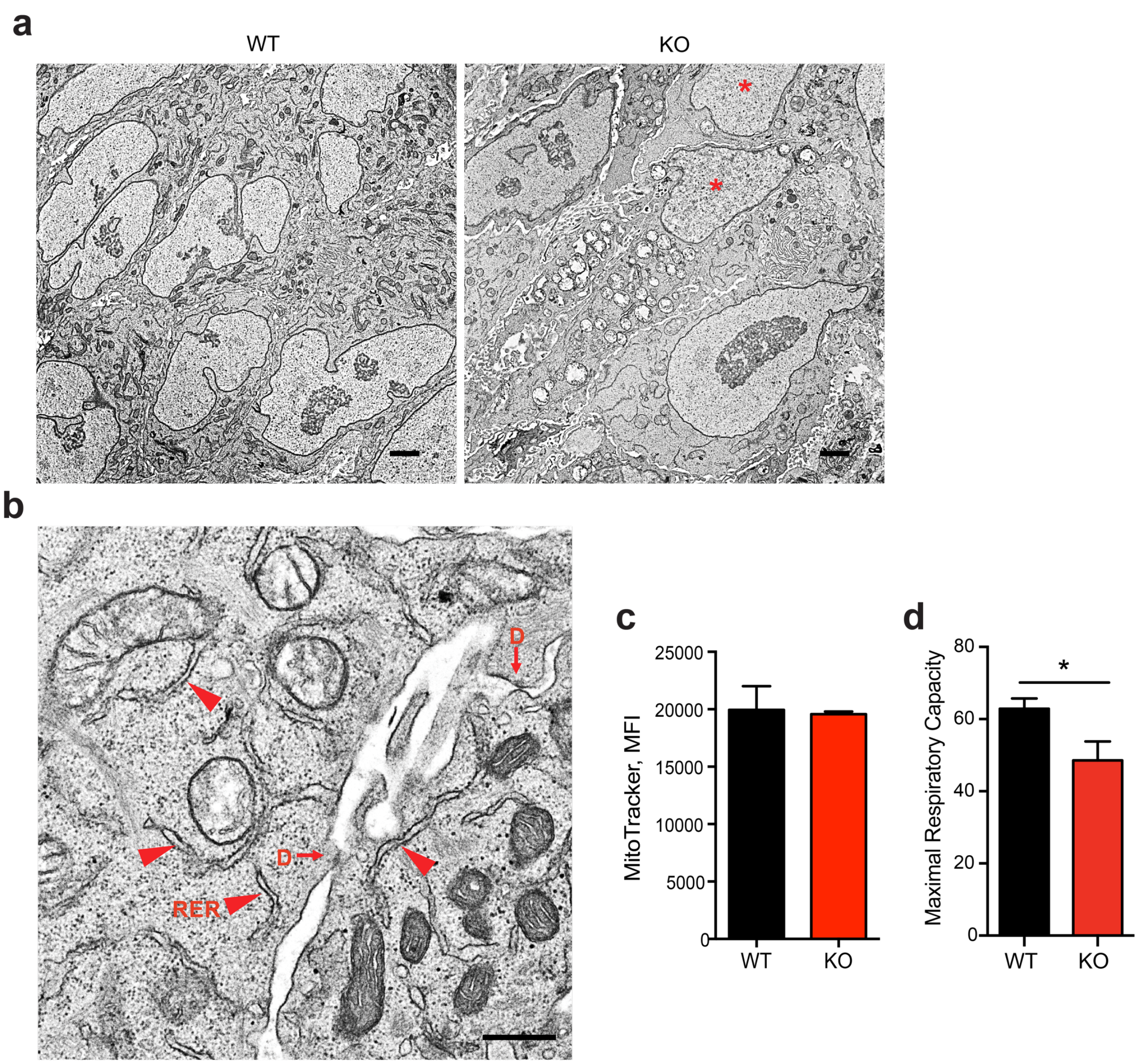

Supplementary Figure 3. Structural and functional defects in mitochondria caused by *TOP1MT* loss. **a** Low magnification electron microscopy images of WT and *TOP1MT*-KO tumor cells corresponding to the enlarged images of mitochondria shown in Fig. 3a. Scale bar, 2 µm. **b** Representative electron micrograph of two neighboring cells in *TOP1MT*-KO xenograft tumors. The swollen mitochondria were analyzed only in nuclei-containing cells with intact cytoplasmic and nuclear membranes and preserved integrity of cytoplasmic organelles. Arrowhead, rough endoplasmic reticulum (RER); thin arrow, desmosome (D); asterisks, cells with swollen mitochondria. Scale bar, 2 µm. **c** Mitochondrial mass determined by Mitotracker Deep Red FM staining of HCT116 WT and *TOP1MT* KO cells (n=3, median ± SEM is plotted). **d** Decreased maximal respiratory activity in *TOP1MT*-KO xenograft tumors measured by oxygen consumption rate (OCR) using the Seahorse BioFlux Analyzer after FCCP treatment and normalized to DNA content (CyQUANT, Invitrogen). All data are means ± SEM unless otherwise stated; *$p<0.05$, Student's *t*-test.

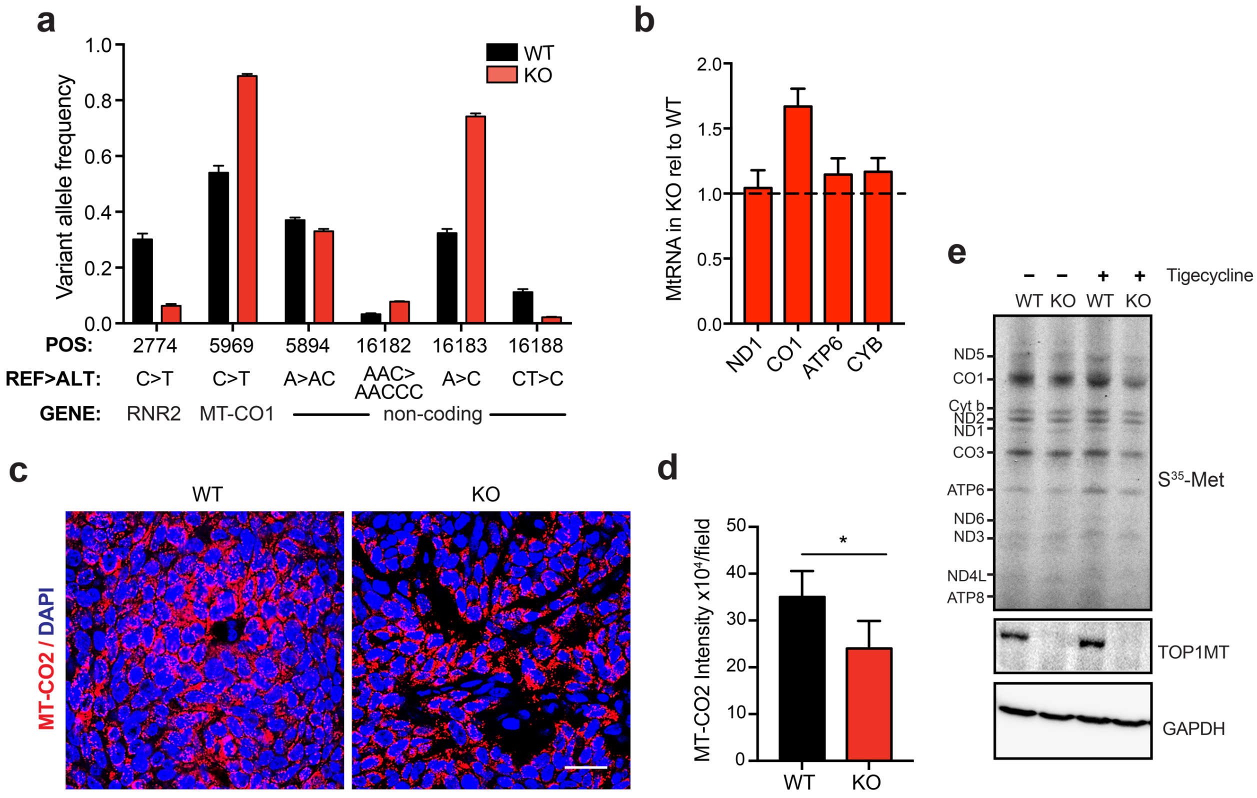

Supplementary Figure 4. mtDNA alterations and respiratory chain protein MT-CO2 levels in WT and *TOP1MT* KO xenograft tumors. **a** Alterations in mtDNA sequence revealed by mitoRCA-seq run on a HiSeq2000 and analyzed using MToolbox. Significant base alterations between WT and KO xenograft tumors with a variant allele frequency ≥ 5% are shown (n=5 tumors for each genotype). The mutation in the *MT-CO1* gene is a synonymous alteration. POS, position in the mitochondrial genome; REF, reference sequence; ALT, alternated sequence. **b** Transcript levels of selected mitochondrial-encoded genes (n=4, each genotype). **c** Representative images of immunofluorescence staining for MT-CO2. Scale bar, 20 µm. **d** Quantification of the immunofluorescence intensity of MT-CO2 in the xenograft tumor sections. **e** Mitochondrial translation determined by autoradiography of S^{35}-methionine incorporation in HCT116 WT and *TOP1MT* KO cells. GAPDH was used as a loading control. The data are means ± SEM; *p<0.05, Student's *t*-test.

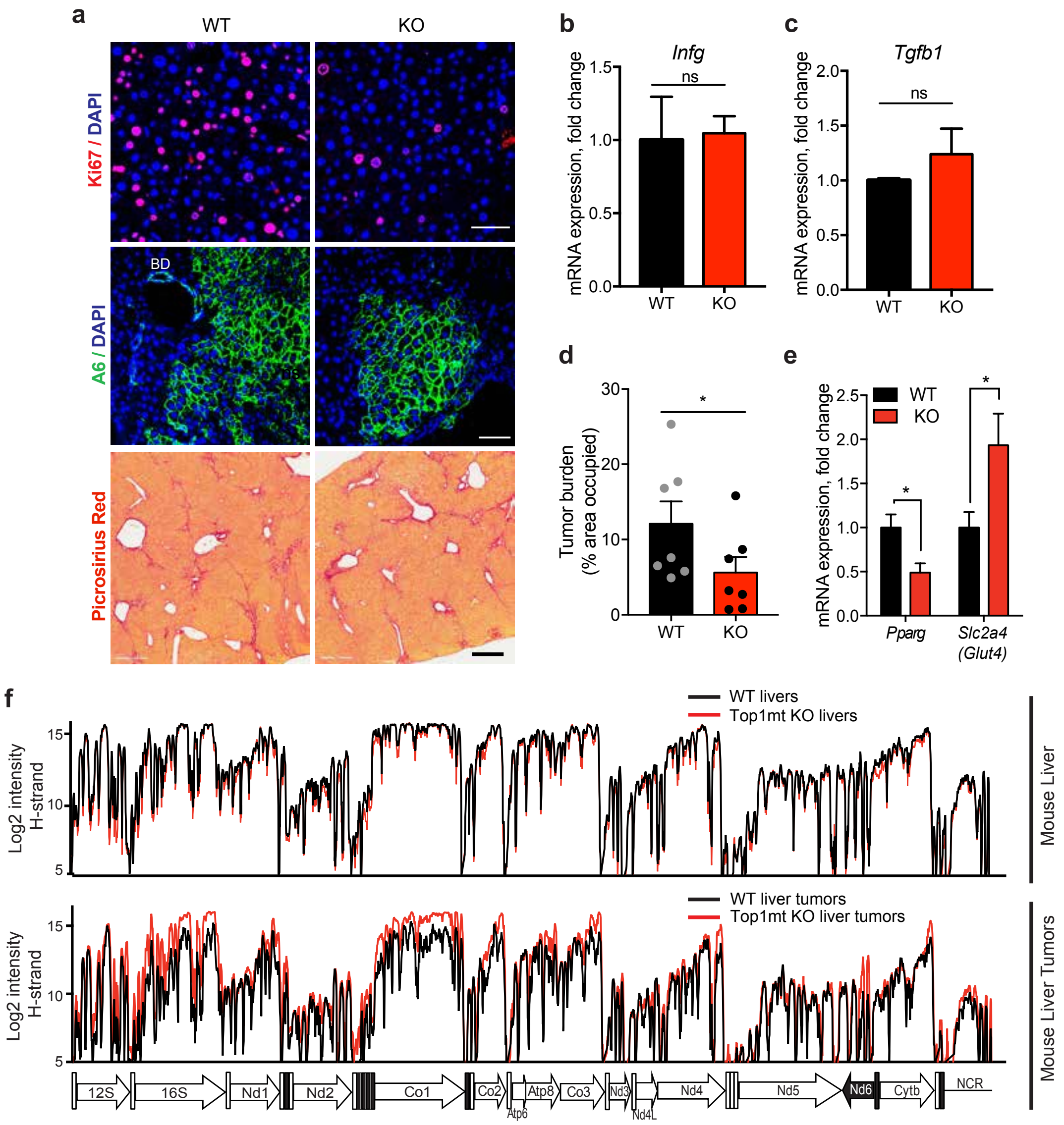

Supplementary Figure 5. Gene expression analysis, immunofluorescence staining and mitochondrial transcript profiles in drug-induced tumors developed in WT and *Top1mt* KO mice. **a** Representative images of immunofluorescence staining for proliferation marker Ki67 (top), hepatic progenitor marker A6 (middle), and fibrosis marker Picrosirius red (bottom). The majority of liver tumors from both WT and *Top1mt*-/- mice displayed a positive staining with a hepatic progenitor marker A6[1]. Nuclei were counterstained with DAPI. Scale bars, 50 µm (top and middle), 300 µm (bottom). BD, bile duct. **b** and **c** Gene expression of *Infg* (**b**) and *Tgfb1* (**c**) in the surrounding liver of WT and *Top1mt* KO mice determined by qPCR (n=3 animals, each performed in duplicates). **d** Quantification of tumor burden 50 weeks after a single intraperitoneal injection of diethylnitrosamine (25 mg/kg body weight) was given 14 days after birth. Tumor burden is expressed as proportion of hepatic parenchyma occupied by tumor tissue on H&E sections, n=7 animals per genotype. **e** qPCR analysis of the indicated genes (n=5, each performed in duplicates). **f** Mitochondrial transcription profiles of the heavy strand of normal mouse livers (top panel) and DEN/CCl_4-induced HCCs (bottom panel) using a tiling array specific for mtDNA, n=4 for each tissue type and genotype.

The data are means ± SEM; ns, not significant; *$p<0.05$, Student's *t*-test.

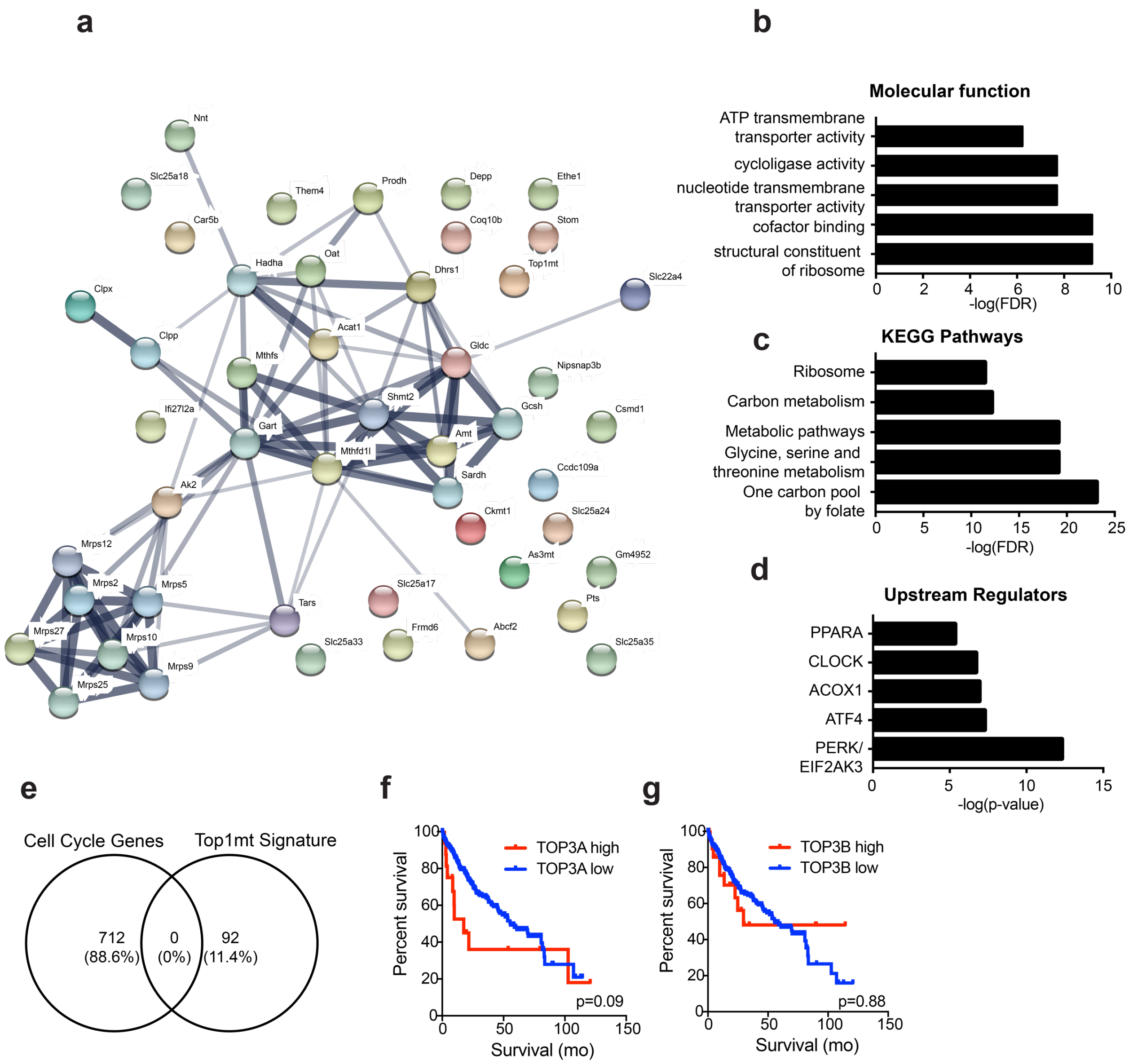

Supplementary Figure 6. Molecular functions and pathways identified by STRING pathway and Ingenuity pathway analyses in murine HCCs induced by a DEN/CCl_4 treatment (related to Figure 6). **a** STRING network analysis of mitochondrial differentially regulated genes in *Top1mt* KO liver tumors compared to WT tumors. **b-c** STRING pathway analysis of molecular functions and differentially regulated KEGG pathways based on the RNA-Seq data of murine HCC tumors (n=3 for each genotype). **d** Ingenuity pathway analysis of upstream regulators in *Top1mt*-deficient liver tumors. **e** Venn diagram of the overlap of the determined *Top1mt* gene expression signature with a curated consensus catalog of cell cycle regulated genes. **f-g** Survival rate of patients with HCC distinguished by TOP3α (**f**, 371 total cases from the TCGA database including 18 patients with high *TOP3A* expression, 4.8%) or TOP3β expression (**g**, 371 total cases from the TCGA database including 22 patients with high *TOP3B* expression, 5.9%).

a Related to Supplementary Fig. 2c

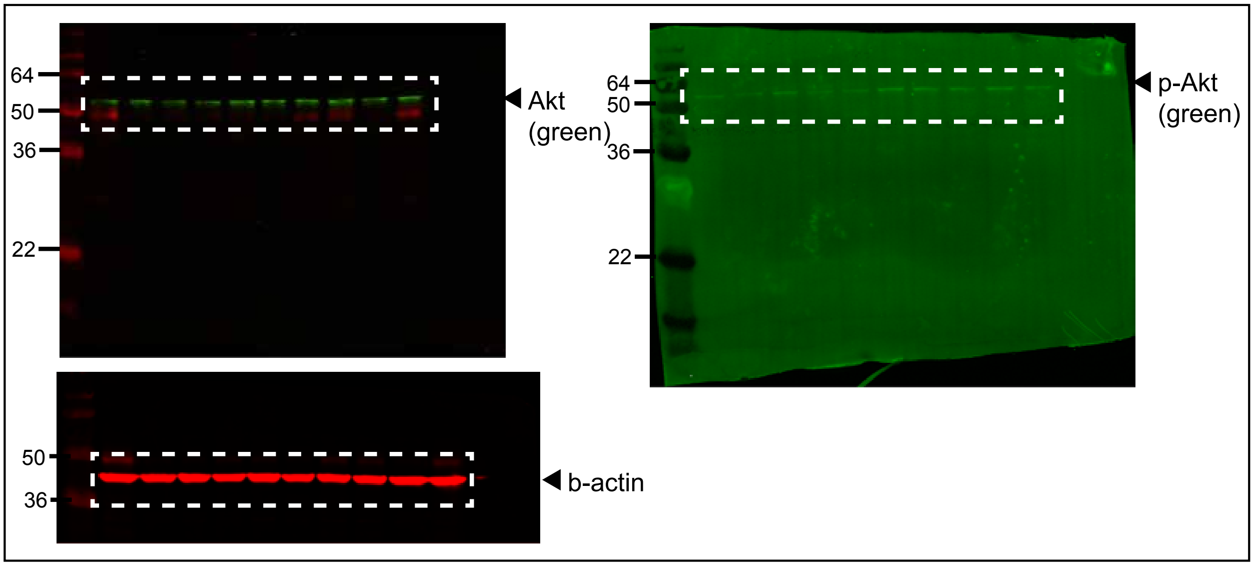

b Related to Fig. 4c

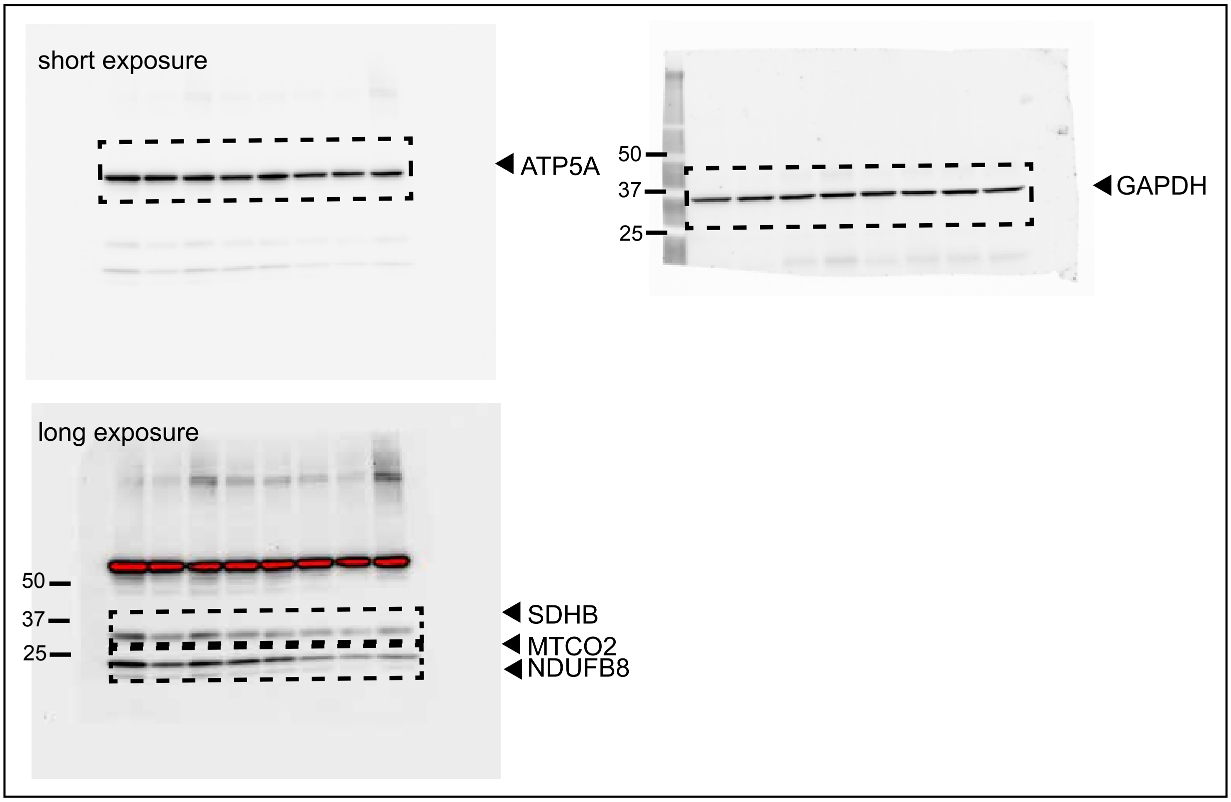

c Related to Fig. 4f

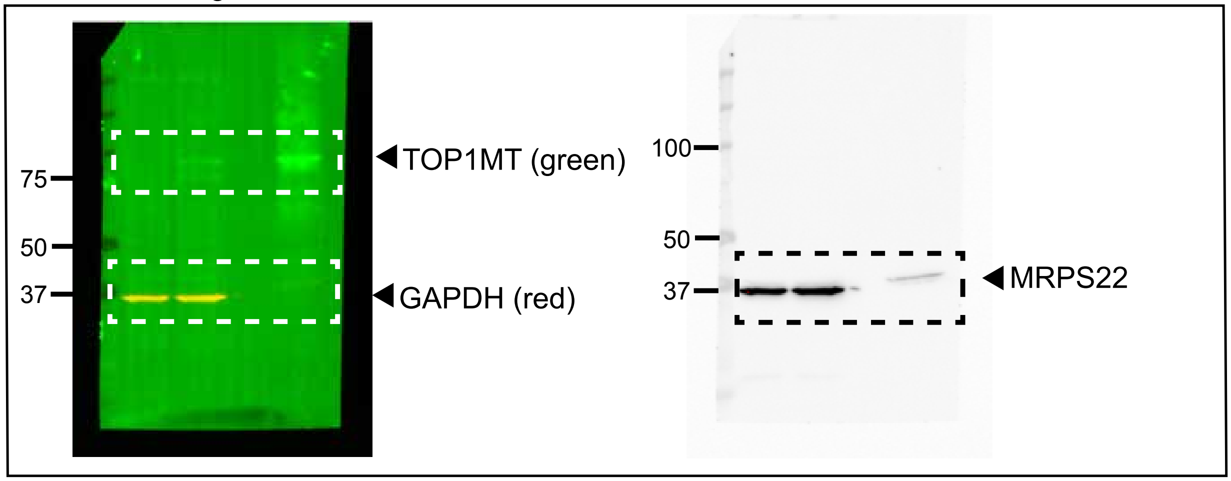

d Related to Fig. 4g

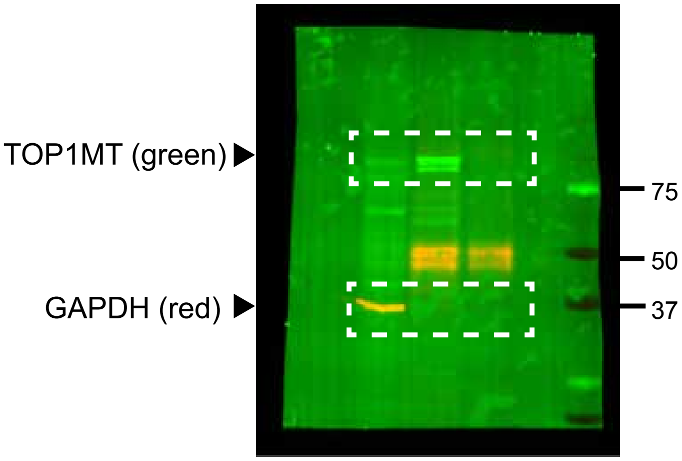

e Related to Supplementary Fig. 4e

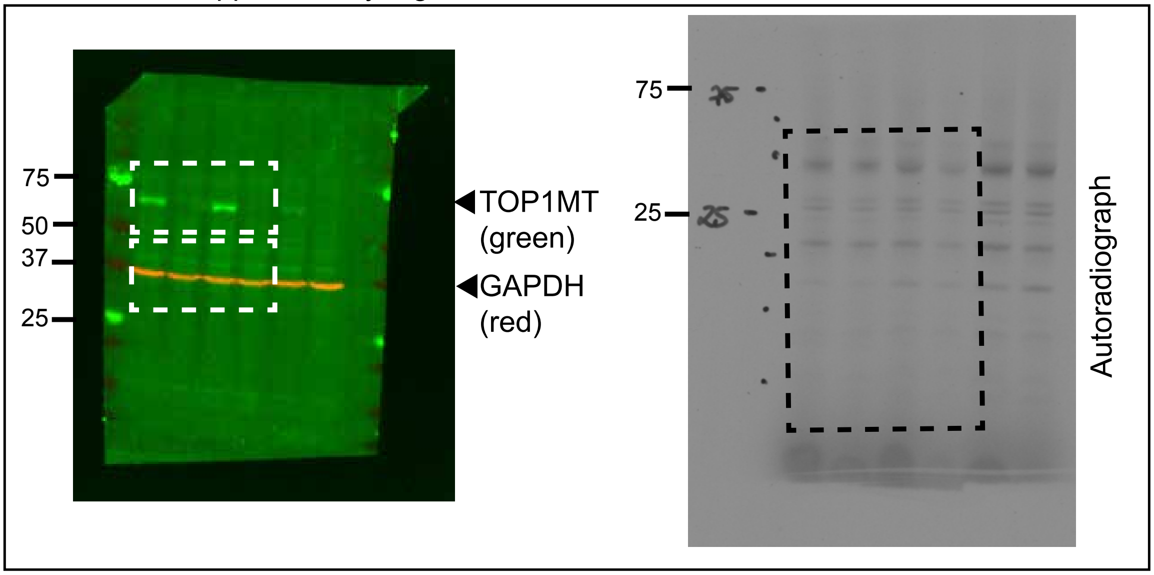

f Related to Fig. 5j

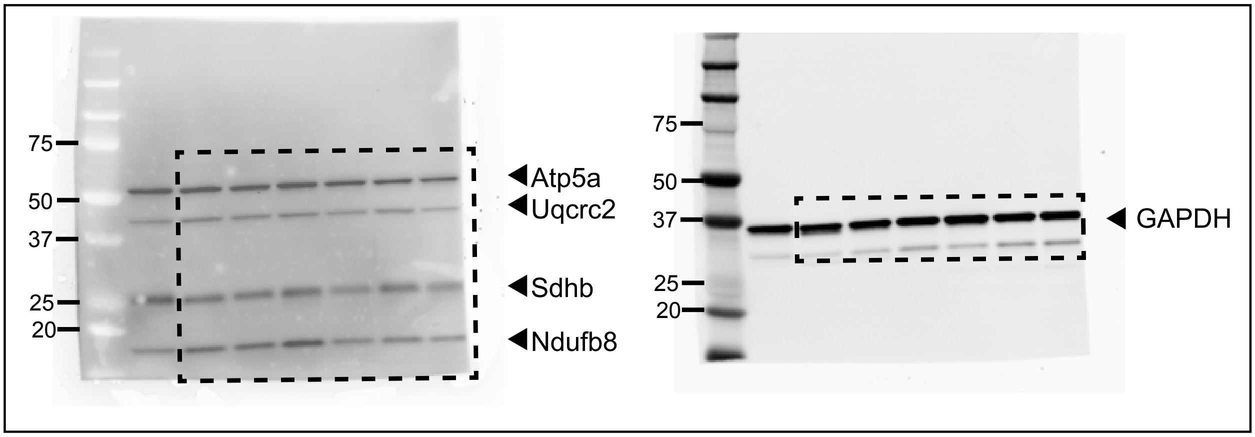

Supplementary Figure 7. Uncropped Western Blot images and gel scans. **a** Supplementary Figure 2c, **b** Figure 4c. **c** Figure 4f. **d** Figure 4g. **e** Supplementary Figure 4e. **f** Figure 5j.

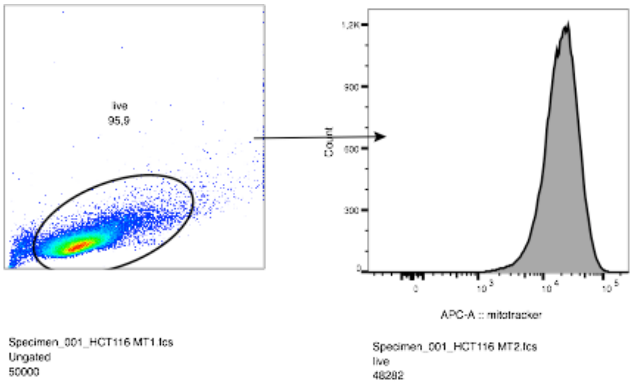

Supplementary Figure 8 Gating Strategy for MitoTracker Deep Red staining.

Supplementary Table 1: Functional associations of significantly altered genes in xenograft tumors determined by STRING pathway analysis. Significantly altered genes in WT and *TOP1MT*-KO xenograft tumors were detected using the nCounter PanCancer Progression panel from NanoString Technologies; FDR, False Discovery Rate; Upregulated and downregulated genes are marked in red and blue, respectively.

Pathway ID	Pathway description	Gene count	FDR	Matching proteins in network (labels)
4151	PI3K-Akt signaling pathway	15	9.08E-10	AKT2, AKT3, FGFR2, FGFR4, FN1, ITGB7, LAMA4, MAPK3, MET, MYC, PDGFC, PRKAA2, SYK, VEGFB, VEGFC
5200	Pathways in cancer	14	3.38E-09	AKT2, AKT3, BMP4, CDKN2A, FGFR2, FN1, LAMA4, MAPK3, MET, MYC, PPARG, RBX1, VEGFB, VEGFC
4510	Focal adhesion	11	3.66E-08	AKT2, AKT3, FN1, ILK, ITGB7, LAMA4, MAPK3, MET, PDGFC, VEGFB, VEGFC
4066	HIF-1 signaling pathway	8	4.83E-07	AKT2, AKT3, CAMK2D, HKDC1, MAPK3, PFKFB1, RBX1, TIMP1
4015	Rap1 signaling pathway	9	5.90E-06	AKT2, AKT3, FGFR2, FGFR4, MAPK3, MET, PDGFC, VEGFB, VEGFC

Supplementary Table 2: Antibodies used in the study

Antibody	Company	Catalog Number	Dilution
A6	DSHB	A6 BCM-s	1:50
Akt	Cell Signaling Technology	4691S	1:1000
b-actin	Sigma-Aldrich	A5441	1:2000
Cleaved caspase 3	Cell Signaling Technology	9661	1:100
GAPDH	Cell Signaling Technology	5174	1:2000
IgG	Santa Cruz	sc-2027	4 μg
IRDye 800CW (goat anti-mouse)	Licor Biosciences	926-32210<br>Lot C61012-05	1:2000
IRDye 680 RD (goat anti-rabbit)	Licor Biosciences	926-68071<br>Lot C70901-15	1:2000
Ki67	abcam	ab16667	1:100
MRPS22	Thermo Fisher Scientific	PA5-52249	1:1000
MTCO2	Thermo Fisher Scientific	MS-1372-P1	1:200
OXPHOS	abcam	ab110411	1:1000
OXPHOS Rodent	abcam	ab110413	1:1000
p-Akt	Cell Signaling Technology	9271S	1:500
TOP1MT	DSHB	CPTC-TOP1-MT-3	1:250

Supplementary Table 3: Primer list

**Primer**	**Sequence**	
β2M F	5′-TGCTGTCTCCATGTTTGATGTATCT-3′	
β2M R	5′-TCTCTGCTCCCCACCTCTAAGT-3′	
ND1 F	5′-AAGTCACCCTAGCCATCATTCTAC-3′	
ND1 R	5′-GCAGGAGTAATCAGAGGTGTTCTT-3′	
**Commercial primer**		
	**Company**	**Catalog Number**
PI3KR1	Applied Biosystems, Taqman Assay	Hs00933163_m1
AKT3	Applied Biosystems, Taqman Assay	Hs00987350_m1
HK	Applied Biosystems, Taqman Assay	Hs00228405_m1
GAPDH	Applied Biosystems, Taqman Assay	Hs02786624_g1
Slc2a1	Applied Biosystems, Taqman Assay	Mm00441480_m1
Top1mt	Applied Biosystems, Taqman Assay	Mm01205855_m1
Pparg	Applied Biosystems, Taqman Assay	Mm00440940_m1
Infg	Integrated DNA Technologies	Mm.PT.58.41769240
Tgfb1	Integrated DNA Technologies	Mm.PT.58.11254750
Gapdh	Applied Biosystems, Taqman Assay	Mm99999915 g1
B2m	Integrated DNA Technologies	Mm.PT.39a.22214835

Supplementary Reference

1. Factor VM, Radaeva SA, Thorgeirsson SS. Origin and fate of oval cells in dipin-induced hepatocarcinogenesis in the mouse. *The American journal of pathology* **145**, 409-422 (1994).
