# Supplementary material for: The mitochondrial type IB topoisomerase drives mitochondrial translation and carcinogenesis
Source: Nat Commun. 2019 Jan 8;10:83. doi: 10.1038/s41467-018-07922-3 (PMC6325124; doi:10.1038/s41467-018-07922-3)
Supplement: Supplementary file 5 — Description of Additional Supplementary Files [file 41467_2018_7922_MOESM5_ESM.docx]

**Title:** Supplementary Data 1:
**Description:** List of significantly altered genes in WT and TOP1MT-KO xenograft tumors (n=4 for each genotype) determined by nCounter PanCancer Progression panel from NanoString Technologies.

**Title:** Supplementary Data 2:
**Description:** List of interacting proteins with TOP1MT identified by immunoprecipitation followed by mass spectrometry.

**Title:** Supplementary Data 3:
**Description:** List of significantly altered genes in DEN/CCl4 murine HCCs determined by RNA-Seq analysis (n=3 for each genotype).
