# Supplementary material for: The mitochondrial type IB topoisomerase drives mitochondrial translation and carcinogenesis
Source: Nat Commun. 2019 Jan 8;10:83. doi: 10.1038/s41467-018-07922-3 (PMC6325124; doi:10.1038/s41467-018-07922-3)
Supplement: Supplementary file 6 — Reporting Summary [file 41467_2018_7922_MOESM6_ESM.pdf]

nature research

Corresponding author(s): Yves Pommier

Reporting Summary

Nature Research wishes to improve the reproducibility of the work that we publish. This form provides structure for consistency and transparency in reporting. For further information on Nature Research policies, see Authors & Referees and the Editorial Policy Checklist.

Statistical parameters

When statistical analyses are reported, confirm that the following items are present in the relevant location (e.g. figure legend, table legend, main text, or Methods section).

n/a	Confirmed	
☐	☒	The exact sample size (*n*) for each experimental group/condition, given as a discrete number and unit of measurement
☐	☒	An indication of whether measurements were taken from distinct samples or whether the same sample was measured repeatedly
☐	☒	The statistical test(s) used AND whether they are one- or two-sided<br>*Only common tests should be described solely by name; describe more complex techniques in the Methods section.*
☐	☒	A description of all covariates tested
☐	☒	A description of any assumptions or corrections, such as tests of normality and adjustment for multiple comparisons
☐	☒	A full description of the statistics including central tendency (e.g. means) or other basic estimates (e.g. regression coefficient) AND variation (e.g. standard deviation) or associated estimates of uncertainty (e.g. confidence intervals)
☐	☒	For null hypothesis testing, the test statistic (e.g. *F*, *t*, *r*) with confidence intervals, effect sizes, degrees of freedom and *P* value noted<br>*Give P values as exact values whenever suitable.*
☒	☐	For Bayesian analysis, information on the choice of priors and Markov chain Monte Carlo settings
☐	☒	For hierarchical and complex designs, identification of the appropriate level for tests and full reporting of outcomes
☐	☒	Estimates of effect sizes (e.g. Cohen's *d*, Pearson's *r*), indicating how they were calculated
☐	☒	Clearly defined error bars<br>*State explicitly what error bars represent (e.g. SD, SE, CI)*

Our web collection on statistics for biologists may be useful.

Software and code

Policy information about availability of computer code

Data collection	RNA-Seq, mitoRCA-seq see Method section.
Data analysis	Data for mitoRCA-seq were analyzed using the MToolBox pipeline utilizing the computational resource of the NIH HPC Biowulf cluster (Method section for mitoRCA-seq).<br>Analysis of RNA-Seq data was performed using R programing language and related packages. The output matrix from featureCounts was input into the Bioconductor package DESeq2 for differential expression analysis (detailed description with references in the Method section for RNA-Seq).

For manuscripts utilizing custom algorithms or software that are central to the research but not yet described in published literature, software must be made available to editors/reviewers upon request. We strongly encourage code deposition in a community repository (e.g. GitHub). See the Nature Research guidelines for submitting code & software for further information.

April 2018

Data

Policy information about availability of data

All manuscripts must include a data availability statement. This statement should provide the following information, where applicable:
- Accession codes, unique identifiers, or web links for publicly available datasets
- A list of figures that have associated raw data
- A description of any restrictions on data availability

The data sets for the RNA-seq are available from GEO under Accession code GSE122489.

Field-specific reporting

Please select the best fit for your research. If you are not sure, read the appropriate sections before making your selection.

☒ Life sciences ☐ Behavioural & social sciences ☐ Ecological, evolutionary & environmental sciences

For a reference copy of the document with all sections, see nature.com/authors/policies/ReportingSummary-flat.pdf

Life sciences study design

All studies must disclose on these points even when the disclosure is negative.

Sample size	No statistical methods were used to predetermine sample size for in vitro analysis. At least 3 independent biological experiments were conducted. For the xenograft study, HCT116 WT# cells were transplanted in only 3 animals in the spirit of the 3 R's of animal research, as this group only confirmed our WT results. For all other animal experiments, power calculation indicated that seven to eleven mice per group were required to obtain a statistical power of 0.8.
Data exclusions	We excluded miss-injected mice in the xenograft study.
Replication	To allow sufficient material for tissue collection, the xenograft study was repeated several times, using a new cohort of mice followed by cell transplantation, giving very consistent results. Hepatocarcinogenesis was induced in several litters over time to achieve 10 animals for each genotype. Biochemical experiments were replicated at least three times as described throughout the paper and in the Method section.
Randomization	The experiments were not randomized.
Blinding	In general, the investigators were not blinded to allocation during experiments and outcome assessment.

Reporting for specific materials, systems and methods

Materials & experimental systems

n/a	Involved in the study	
☐	☒	Unique biological materials
☐	☒	Antibodies
☐	☒	Eukaryotic cell lines
☒	☐	Palaeontology
☐	☒	Animals and other organisms
☒	☐	Human research participants

Methods

n/a	Involved in the study	
☒	☐	ChIP-seq
☐	☒	Flow cytometry
☒	☐	MRI-based neuroimaging

Unique biological materials

Policy information about availability of materials

Obtaining unique materials	There are no restrictions.

Antibodies

Antibodies used	All antibodies are listed in Supplementary Table 5 with supplier name, catalog number and dilution used.

Validation	The TOP1MT antibody was validated on human CRISPR KO cells. The specificity of the A6 antibody has been established in several publications (most recent: Kitade et al., Genes Dev, 2013). All other antibodies are validated by the distributor.

Eukaryotic cell lines

Policy information about cell lines

Cell line source(s)	HCT116 cells were obtained from the NCI Development Therapeutics Program.
Authentication	Cell Line Authentication was carried out using Short Tandem Repeat Analysis at Frederick National Laboratory.
Mycoplasma contamination	Cells were routinely tested for mycoplasma by MycoAlert (Lonza) and found negative.
Commonly misidentified lines (See ICLAC register)	No commonly misidentified cell line was used.

Animals and other organisms

Policy information about studies involving animals; ARRIVE guidelines recommended for reporting animal research

Laboratory animals	Athymic Ncr-nu/nu female mice (NCI-Frederick) were used for the xenograft study at 5 weeks of age (Fig. 1). Tumor growth was then followed for a maximum of 35 days. For the hepatocarcinogenesis study (Fig. 5) male C57Bl/6 WT and Top1mt KO mice were used. Mice were sacrificed at 22 weeks (DEN and CCl4 treatment group) or at 52 weeks (DEN group).
Wild animals	No wild animals were used in this study.
Field-collected samples	No field-collected samples were used in this study.

Flow Cytometry

Plots

Confirm that:

☒ The axis labels state the marker and fluorochrome used (e.g. CD4-FITC).

☒ The axis scales are clearly visible. Include numbers along axes only for bottom left plot of group (a 'group' is an analysis of identical markers).

☒ All plots are contour plots with outliers or pseudocolor plots.

☒ A numerical value for number of cells or percentage (with statistics) is provided.

Methodology

Sample preparation	Cells (300,000) were seeded in 6-well plates and allowed to grow for 24 h. Cells were then incubated with 100 nM MitoTracker Deep Red dye (Thermo Fisher) for 20 min in normal culture medium at 37°C. After three washes with 3 mL warm PBS, cells were trypsinized and resuspended in PBS supplemented with 1% FBS and 2 mM EDTA and immediately analyzed by flow cytometry (BD LSR Fortessa).
Instrument	BD Fortessa LSR
Software	Data were collected using the BD FACSDiva Software (BD Biosciences) and analyzed using FlowJo 10.4.2 software (FlowJO LLC) and Excel. Data was plotted and statistically analyzed in Prism7 (GraphPad).
Cell population abundance	No sorting was performed.
Gating strategy	The gating strategy is shown in Supplementary Figure 8.

☒ Tick this box to confirm that a figure exemplifying the gating strategy is provided in the Supplementary Information.
